# The effect of acupuncture on blood glucose control in patients with type 2 diabetes: a systematic review and meta-analysis of randomized controlled trials

**DOI:** 10.3389/fendo.2025.1596062

**Published:** 2025-06-11

**Authors:** Yuqi Si, Jiayao Chen, Lizhu Chen, Yan Zheng, Yixuan Qiu, Beibei Wang, Yiwen Liang, Yubo Zhang, Yaling Chen

**Affiliations:** ^1^ School of Traditional Chinese Medicine, Hunan University of Chinese Medicine, Changsha, Hunan, China; ^2^ College of Integrated Traditional Chinese and Western Medicine, Hunan University of Chinese Medicine, Changsha, Hunan, China; ^3^ School of Medical, Hunan University of Chinese Medicine, Changsha, Hunan, China; ^4^ School of Humanities and Management, Hunan University of Chinese Medicine, Changsha, Hunan, China

**Keywords:** acupuncture, Traditional Chinese Medicine, T2DM, systematic review, meta-analysis

## Abstract

**Purpose:**

This meta-analysis aimed to ascertain the effectiveness of acupuncture in treating clinical symptoms of type 2 diabetes mellitus (T2DM) and to summarize the acupoints and meridians involved.

**Methods:**

PubMed, Web of Science, Cochrane Library, Embase, China National Knowledge Infrastructure (CNKI), and Wanfang were thoroughly retrieved to acquire randomized controlled trials (RCTs) evaluating acupuncture as an adjunct treatment for T2DM. Outcome measures focused on improvements in T2DM clinical symptoms. The meta-analysis was implemented leveraging RevMan 5.4 and Stata 15 software, with sensitivity and subgroup analyses to assess the stability of results and identify heterogeneity sources.

**Results:**

21 RCTs encompassing 2,117 individuals with T2DM were analyzed. The results of 2-hour postprandial glucose (2h PG), body mass index (BMI), fasting blood glucose (FBG), high-density lipoprotein (HDL), low-density lipoprotein (LDL), bilateral median nerve motor conduction velocity, and plasma viscosity were reliable. No publication bias was noted, except for Packed Cell Volume (PCV) and Traditional Chinese Medicine Syndrome Score Scale (TCMSS). The meta-analysis showed that acupuncture significantly improved clinical markers such as glycated hemoglobin A1c (HbA1c), 2h PG, FBG, and fasting serum insulin (FINS). Subgroup analysis for FBG, 2h PG, and triglycerides (TG) indicated that the primary source of heterogeneity for FBG was related to participants with uncomplicated T2DM and a treatment duration of less than three months. No significant heterogeneity was observed for 2h PG, while the TG data were unstable.

**Conclusion:**

Acupuncture can significantly alleviate the main clinical symptoms of T2DM, but significant heterogeneity was observed for individual indicators. Further investigation is needed to corroborate its precise therapeutic effectiveness and identify potential influencing factors.

**Systematic review registration:**

https://www.crd.york.ac.uk/PROSPERO/view/CRD42024602165, identifier CRD42024602165.

## 1 Introduction

Type 2 diabetes mellitus (T2DM) is a chronic metabolic condition marked by either insulin resistance or insufficient insulin secretion, contributing to rapidly elevated blood glucose levels. The prevalence of T2DM is rising globally, and 579 million individuals will be afflicted by 2030 (1). This disease is a prominent contributor to disability and mortality. T2DM-related complications may not only impact the heart, brain, and kidneys, but also cause diabetic retinopathy and diabetic foot, thus considerably compromising the quality of life of affected individuals and creating a considerable societal burden (2).

In conventional medicine, sulfonylureas, biguanides, and insulin are the most frequently prescribed treatments for T2DM. However, these medications often induce side effects (3). Traditional Chinese Medicine (TCM), particularly acupuncture and herbal treatments, has demonstrated encouraging clinical results. Recent studies indicate that acupuncture may help regulate blood glucose levels by enhancing the balance between the sympathetic and parasympathetic nervous systems, as well as modulating the endocrine system (4). Although several studies have unraveled the clinical effectiveness of acupuncture in controlling blood glucose in individuals with T2DM, there remains no consensus. A clinical trial by Liu Xiang (5) showed that acupuncture notably reduced 2-hour postprandial glucose (2h PG) levels, though it had no significant effect on fasting blood glucose (FBG). In contrast, a study by Gaoguo Luo et al. (6) demonstrated that acupuncture effectively lowered both FBG and 2h PG levels when compared to basic interventions. Given these conflicting results, a systematic review and meta-analysis is warranted to ascertain the benefits of acupuncture in managing blood glucose in individuals with T2DM.

The latest research (7) has probed into the therapeutic effects of acupuncture; however, it is limited by methodological weaknesses, such as a narrow range of outcome measures, small sample sizes, and inadequate assessment of publication bias, and no analysis of related complications. Subsequently, new clinical trials have been published. Hence, this updated systematic review aims to incorporate a wider range of studies, broaden the outcome measures, deeply examine publication bias, and perform detailed subgroup analyses to identify sources of heterogeneity. The goal of this research is to elucidate the clinical benefits of acupuncture in treating T2DM and to determine the most suitable patient populations and treatment protocols through subgroup analysis, thereby offering robust scientific evidence and theoretical support for its clinical application.

## 2 Materials and methods

### 2.1 Protocol and registration

The current research was prospectively registered in the International Prospective Register of Systematic Reviews (PROSPERO) with the registration number CRD42024602165. The meta-analysis was implemented as per the guidelines of the Preferred Reporting Items for Systematic Reviews and Meta-Analyses (PRISMA), as well as its protocol and extension statement (8).

### 2.2 Search strategy

Multiple databases were retrieved, encompassing PubMed, Web of Science, Cochrane Library, Embase, China National Knowledge Infrastructure (CNKI), Wanfang, and other relevant sources to acquire randomized controlled trials (RCTs) evaluating acupuncture as an adjunctive therapy for T2DM, up to January 1, 2025. The search terms used included "Acupuncture, Pharmacopuncture," "Blood Glucose, Blood Sugar, Sugar, Blood, Glucose, Blood," "Diabetes Mellitus, Type 2, Type 2 Diabetes Mellitus, Type 2 Diabetes, T2D, T2DM," and "random." No restrictions were imposed on language or geographic location. Additionally, we manually retrieved the reference lists of the eligible articles to avoid omitting any studies.

### 2.3 Inclusion and exclusion criteria

After evaluating the titles, abstracts, and full texts, RCTs that met the following eligibility criteria were incorporated into the review.

#### 2.3.1 Inclusion criteria

- Participants: Individuals aged 18 years and older, diagnosed with T2DM were eligible for inclusion, with no restrictions on gender or ethnicity.
- Intervention and comparison: The control group underwent standard treatment or conventional Western medicine, while the intervention group received acupuncture along with the treatment provided to the control group.
- Outcomes: The study must provide at least one of the following outcomes. Primary outcomes encompassed insulin sensitivity index (ISI), glycated hemoglobin A1c (HbA1c), 2h PG, insulin, Traditional Chinese Medicine Syndrome Score Scale (TCMSS), and FBG. Secondary outcomes comprised fasting serum insulin (FINS), homeostatic model assessment of insulin resistance (Homa-IR), homeostatic model assessment of beta-cell function (Homa-B), total cholesterol (TC), triglycerides (TG), body weight, body mass index (BMI), body fat percentage, low-density lipoprotein (LDL), high-density lipoprotein (HDL), waist-to-hip ratio (WHR), packed cell volume (PCV), whole blood viscosity, plasma viscosity, fibrinogen (FIB), serum creatinine (Scr), bilateral median nerve motor conduction velocity, and bilateral common peroneal nerve motor conduction velocity.
- Study design: RCT.

#### 2.3.2 Exclusion criteria

- Non-RCTs, retrospective studies, animal research, and reviews.
- Individuals with T2DM induced by factors other than primary diabetes.
- Intervention groups used other TCM treatments, for instance, proprietary Chinese medicines, herbal pills, herbal injections, or massage.
- Studies that did not provide primary outcomes linked to T2DM, had inaccurate data, incomplete outcome measures, or where data could not be obtained from the original authors.
- Duplicate publications.
- Studies published in non-core journals (non-Peking University Core Journals) or Chinese literature from the Chinese Science and Technology Papers and Citations Database (CSTPD) due to quality concerns.
- Studies with an intervention period shorter than 2 weeks.

### 2.4 Data extraction

Two researchers (Yuqi Si and Jiayao Chen) separately extracted relevant data. The extracted information encompassed (a) Publication details: Title, first author, and year of publication; (b) Study characteristics: Study design and duration of treatment; (c) Participant characteristics: The count of participants, age, sex, duration of T2DM, and BMI; (d) Intervention: Medications and dosages used in the control group, frequency, and route of administration; acupuncture points, frequency, duration, and methods used in the intervention group, based on the control group; (e) Outcomes: Primary and secondary outcomes. For continuous data, means and standard deviations were extracted. In terms of categorical data, event counts and total numbers were extracted (**Supplementary Table S1**).

### 2.5 Quality assessment

The risk of bias (RoB) in the eligible RCTs was ascertained by two investigators (Yuqi Si and Lizhu Chen) leveraging the Cochrane risk of bias tool (9), and they cross-checked their results. The evaluation tool covered seven domains: random sequence generation, allocation concealment, blinding of participants and personnel, completeness of outcome data, selective reporting, and other biases. The methodological quality of the included studies was categorized into three groups: "high RoB," "low RoB," and "unclear RoB." In case of any dissent, a third researcher (Jiayao Chen) made the final decision.

### 2.6 Statistical analysis

Literature management was performed using EndNote 9.0; data organization was conducted in Excel; and statistical analyses were executed by employing RevMan 5.4 and Stata 15.0.

For binary outcomes, risk ratio (RR) was utilized as the effect measure, while for continuous variables, the standardized mean difference (SMD) was applied. All effect sizes were reported with their corresponding 95% confidence intervals (CIs). Cochrane Q test and I² test were utilized to probe into heterogeneity. Significant heterogeneity was indicated if P < 0.1 and I² ≥ 50%. A random-effects model was applied for all analyses. Meta-analysis was conducted at a significant level of α = 0.05. Sensitivity analysis was implemented by excluding the studies one by one to ascertain the stability of the outcome measures, and publication bias was ascertained by adopting funnel plots and Egger's test. Additionally, subgroup analysis was executed for outcomes reported in at least 10 studies to explore the stability of the results and identify potential sources of heterogeneity.

## 3 Results

### 3.1 Studies and selection

**Figure 1:**
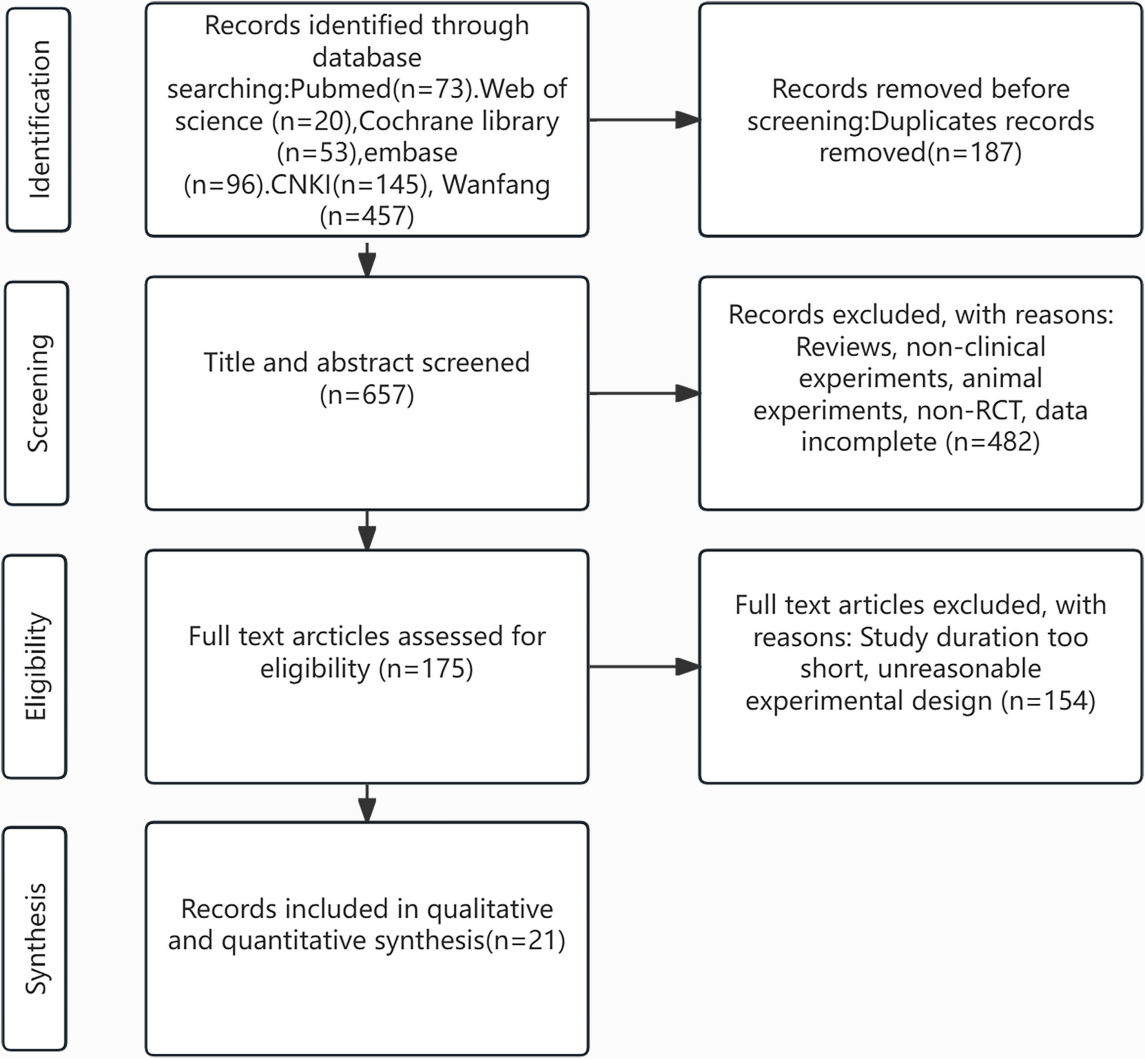
Flow plot.

Initially, 884 relevant studies were acquired from databases. After excluding 187 duplicate publications, 657 studies were screened as per their titles and abstracts, and subsequently, 482 studies were ruled out. The full texts of the remaining 175 studies were checked. Finally, 21 studies were incorporated (5, 6, 10–28). The process of study selection is illustrated in **Figure 1**.

### 3.2 Study characteristics

The 21 studies included in the review involved 2,117 patients, with 1,010 participants in the intervention group and 1,107 in the control group. Both groups had a sample size of more than 10 participants. The patients came from various regions, encompassing China, Brazil, Egypt, Iran, and others. The average age of participants in the intervention group varied from 33.97 to 63.87 years, while in the control group, it ranged from 34.74 to 64.2 years. Treatment durations varied from 2 weeks to 1 year. The patients were diagnosed with different conditions, encompassing simple T2DM, T2DM with complications, T2DM with obesity, and impaired glucose tolerance.

The treatment methods in the control group consisted of conventional treatments, lifestyle modifications, hypoglycemic medications, injectable drugs, and sham acupuncture. In the intervention group, acupuncture was administered in addition to the treatments utilized in the control group. The acupuncture methods comprised electroacupuncture, auricular acupuncture, thread-embedding acupuncture, laser acupuncture, wrist-ankle acupuncture, and body acupuncture. The acupoints were based on the Chinese national standard Acupuncture Point Names and Locations (GB/T 12346–2021) and the WHO International Acupuncture Standard, which defined their codes, therapeutic effects, and anatomical locations.

A total of 59 different acupuncture points from 12 meridians were utilized, along with additional points, auricular acupuncture zones, wrist-ankle acupuncture zones, and hypoglycemic points. The five most frequently utilized acupoints encompassed Zusanli (ST36), Sanyinjiao (SP6), Hegu (LI4), Pishu (BL20), and Quchi (LI11). For patients with T2DM combined with complications, obesity, or prediabetes, acupuncture was predominantly applied to the Stomach Meridian (Foot Yangming) in the lower limb. For those with simple T2DM, acupuncture was performed on the Bladder Meridian (Foot Taiyang). For patients with impaired glucose tolerance, acupuncture was mainly concentrated on areas of the Bladder Meridian (Foot Taiyang). Detailed information on the number of points, anatomical locations, therapeutic effects, and meridian correspondences is summarized in **Supplementary Tables S2**, **S3**.

### 3.3 RoB assessment

The quality of the eligible studies was appraised by leveraging the Cochrane risk of bias tool. All 21 studies adhered to the design of RCTs and were classified as having a low RoB. Eleven studies employed random allocation methods, encompassing random number tables used in eight studies, a drawing method for random allocation in one study (23), and computer-based randomization in one study (25); hence, these studies were rated as having a low risk. Seven studies utilized central randomization with allocation concealment; one study leveraged the envelope method (27); and another applied a drawing method (23); these studies were also rated as low risk. The remaining studies did not report on allocation concealment, which resulted in an unclear risk rating.

Seven studies were rated as having a low RoB in blinding since they properly utilized blinding methods during the intervention phase. One study mentioned single-blinding (23) and was rated as high risk. Another study did not implement blinding (24) and was also rated as high risk. The rest of the studies did not specify whether blinding was used, leading to an unclear RoB. Four studies implemented blinding in the outcome assessment process and thereby were rated as a low RoB. The remaining studies did not describe blinding for outcome assessments, leading to an unclear RoB.

**Figure 2:**
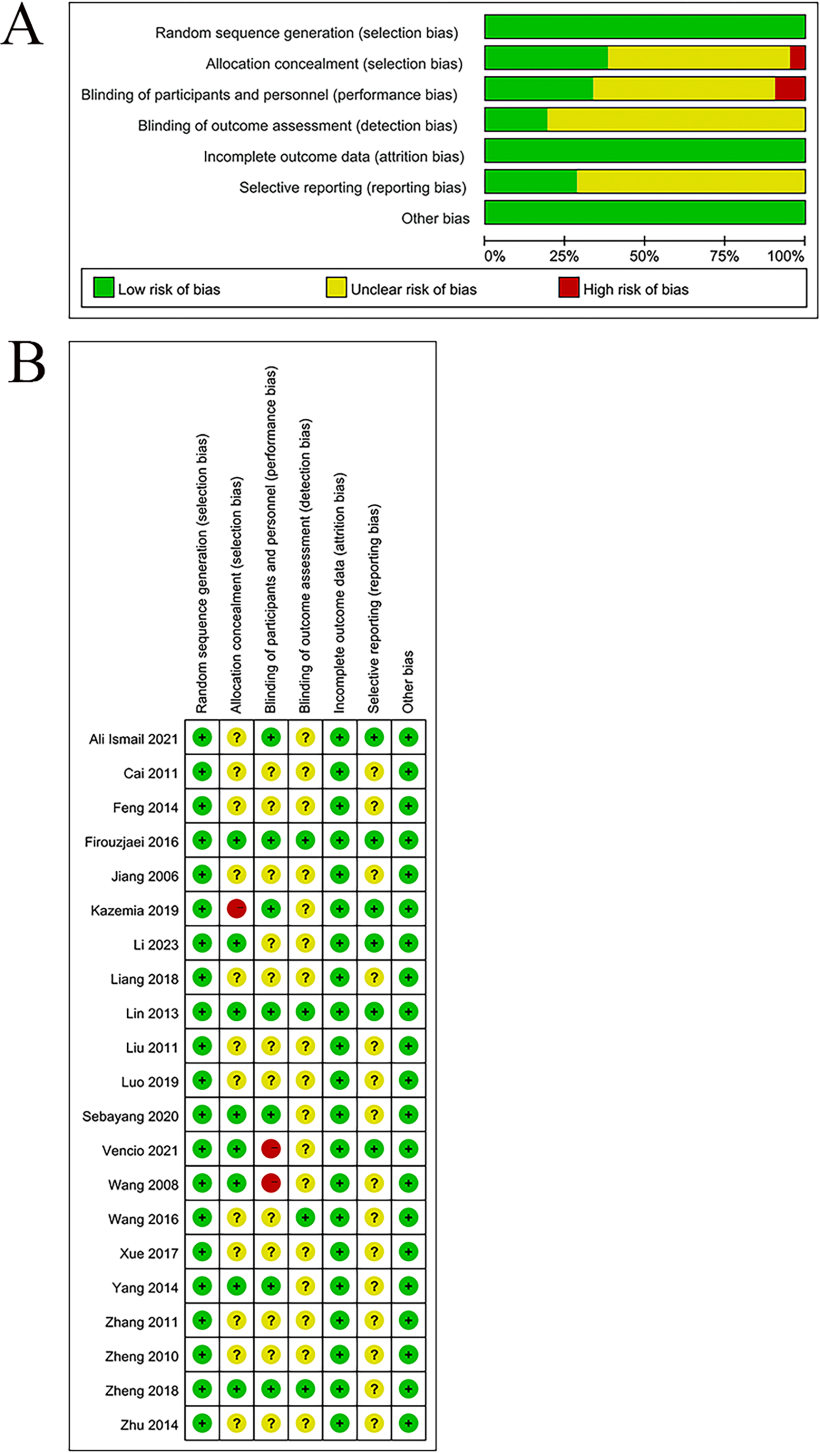
**(A)** Risk of bias graph; **(B)** Risk of bias summary.

All studies reported complete outcome data and were rated as low risk for data completeness. Six studies were registered and showed no selective reporting, and thus they were considered low risk. The other studies did not clarify whether they were registered, and selective reporting could not be assessed, which led to an unclear risk. No additional sources of bias were noted in any of the studies, and all were rated as low risk. The detailed results are provided in **Figures 2A, B**.

### 3.4 Main results

**Figure 3:**
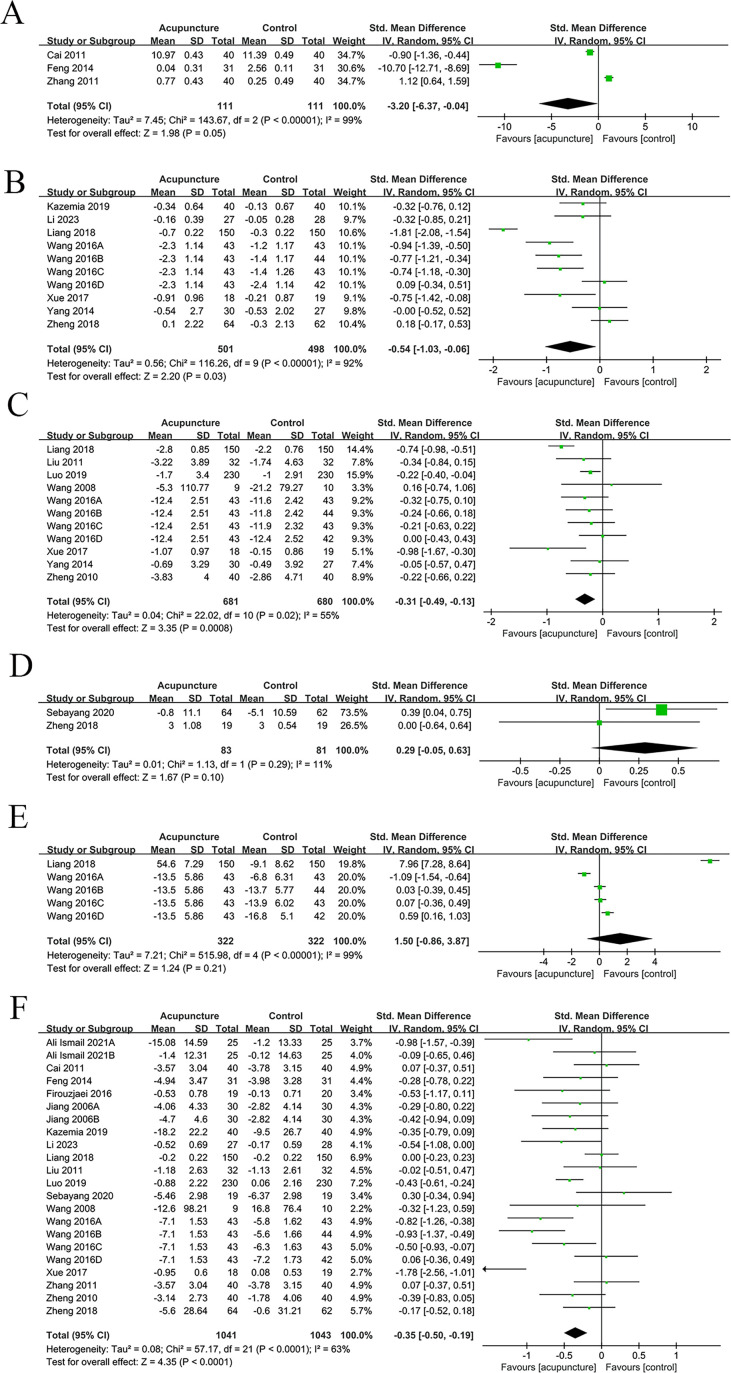
Main results forest plot. **(A)** Forest plot ISI; **(B)** Forest plot Glycated HbA1c; **(C)** Forest plot 2h PG; **(D)** Forest plot Insulin levels; **(E)** Forest plot TCMSS; **(F)** Forest plot FBG.

Three studies reported ISI. The results indicated that acupuncture significantly improved ISI in T2DM patients in comparison to the control group (SMD = -3.20, 95% CI [-6.37, -0.04], I² = 99%, p < 0.00001) (**Figure 3A**).

Ten studies examined HbA1c. The results unraveled that acupuncture considerably ameliorated HbA1c levels in comparison to the control group (SMD = -0.54, 95% CI [-1.03, -0.06], I² = 92%, p < 0.00001) (**Figure 3B**).

Eleven studies focused on 2h PG. The results uncovered that acupuncture significantly improved the 2h PG levels (SMD = -0.31, 95% CI [-0.49, -0.13], I² = 55%, p ≤ 0.02) (**Figure 3C**).

Two studies were meta-analyzed. The results unraveled no considerable difference in insulin levels between the acupuncture and control groups (SMD = 0.29, 95% CI [-0.05, 0.63], I² = 11%, p = 0.29) (**Figure 3D**).

Five studies reported TCMSS scores. No considerable difference was noted in TCMSS scores between acupuncture and control groups (SMD = 1.50, 95% CI [-0.86, 3.87], I² = 99%, p < 0.00001) (**Figure 3E**).

Twenty-two studies reported FBG levels. The results demonstrated that acupuncture considerably ameliorated FBG levels in comparison to the control group (SMD = -0.35, 95% CI [-0.50, -0.19], I² = 63%, p < 0.00001) (**Figure 3F**).

### 3.5 Secondary results

Acupuncture ameliorated several secondary outcomes in individuals with T2DM compared to the control group, encompassing FINS (SMD = -1.09, 95% CI [-1.72, -0.46], I² = 79%, p = 0.0007), Homa-IR (SMD = -0.51, 95% CI [-0.84, -0.17], I² = 0%, p = 0.003), Homa-B (SMD = -0.78, 95% CI [-1.51, -0.05], I² = 93%, p = 0.04), TG (SMD = -0.16, 95% CI [-0.32, -0.01], I² = 26%, p = 0.04), BMI (SMD = -0.36, 95% CI [-0.63, -0.09], I² = 71%, p = 0.009), LDL (SMD = -0.46, 95% CI [-0.79, -0.12], I² = 82%, p = 0.008), HDL (SMD = 0.68, 95% CI [0.29, 1.07], I² = 91%, p = 0.0007), WHR (SMD = 0.77, 95% CI [0.35, 1.19], I²= 0%, p = 0.0003), plasma viscosity (SMD = -0.63, 95% CI [-0.85, - 0.42], I² = 0%, p < 0.00001), bilateral median nerve motor conduction velocity (SMD = 0.69, 95% CI [0.36, 1.02], I² = 0%, p < 0.0001), bilateral common peroneal nerve motor conduction velocity(SMD = 0.38, 95% CI [0.06,0.71], I² = 0%, p = 0.02)

No considerable differences were noted in TC (SMD = -0.30, 95% CI [-0.60, -0.00], I² = 75%, p = 0.05), body weight (SMD = -0.18, 95% CI [-0.57, 0.21], I² = 0%, p = 0.37), body fat percentage (SMD = -0.14, 95% CI [-0.50, 0.22], I² = 0%, p = 0.44), PCV (SMD = -0.37, 95% CI [-0.79, 0.05], I² = 74%, p = 0.09), whole blood viscosity (SMD = -0.18, 95% CI [-0.44, 0.08], I² = 34%, p = 0.17), FIB (SMD = -0.27, 95% CI [-0.59, 0.05], I² = 55%, p = 0.10), and Scr (SMD = -0.46, 95% CI [-1.90, 0.98], I² = 93%, p = 0.53). Detailed information can be found in **Supplementary Figures S1**, **S2**.

### 3.6 Publication bias

**Figure 4:**
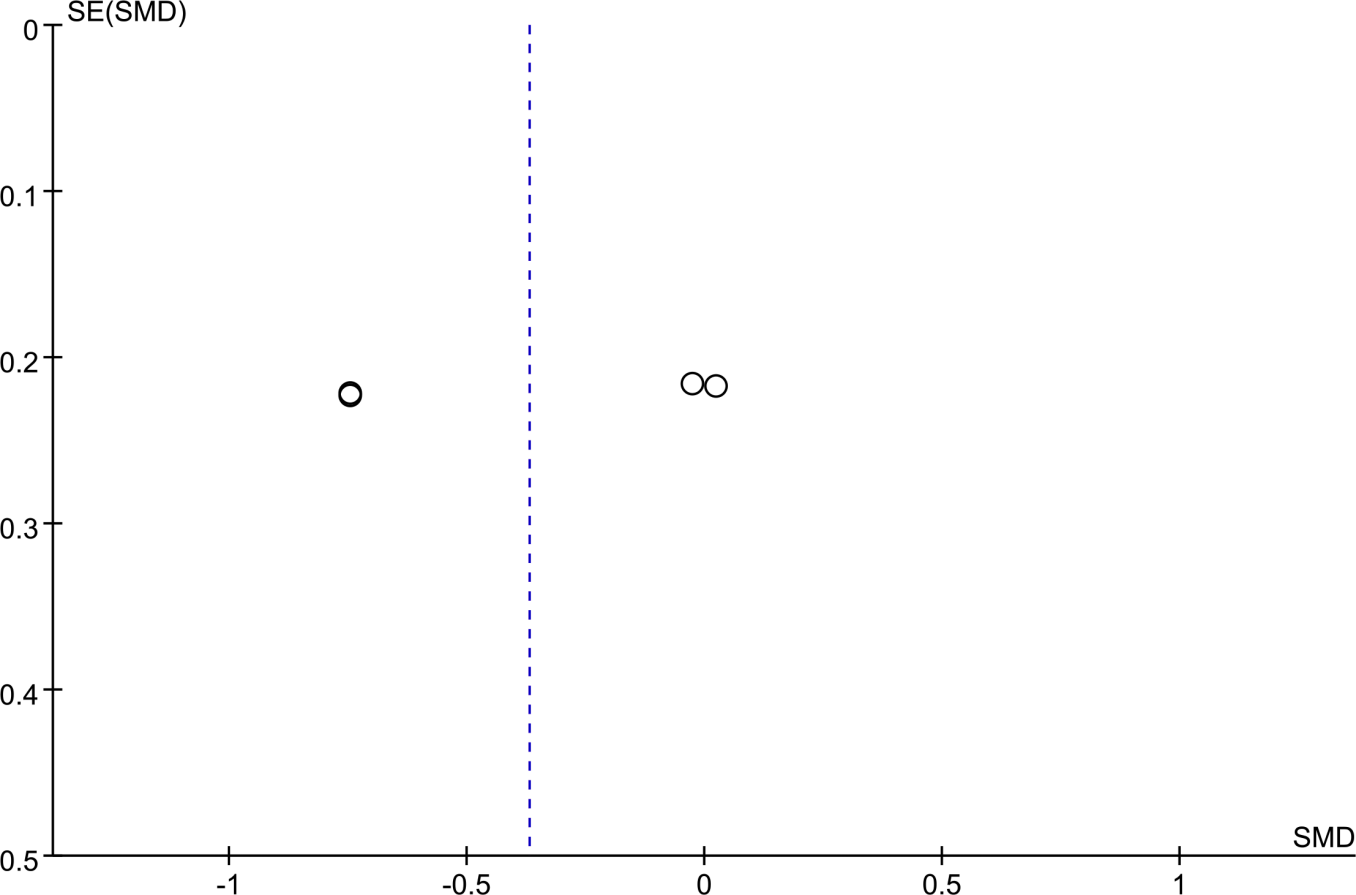
PCV funnel plot.

**Figure 5:**
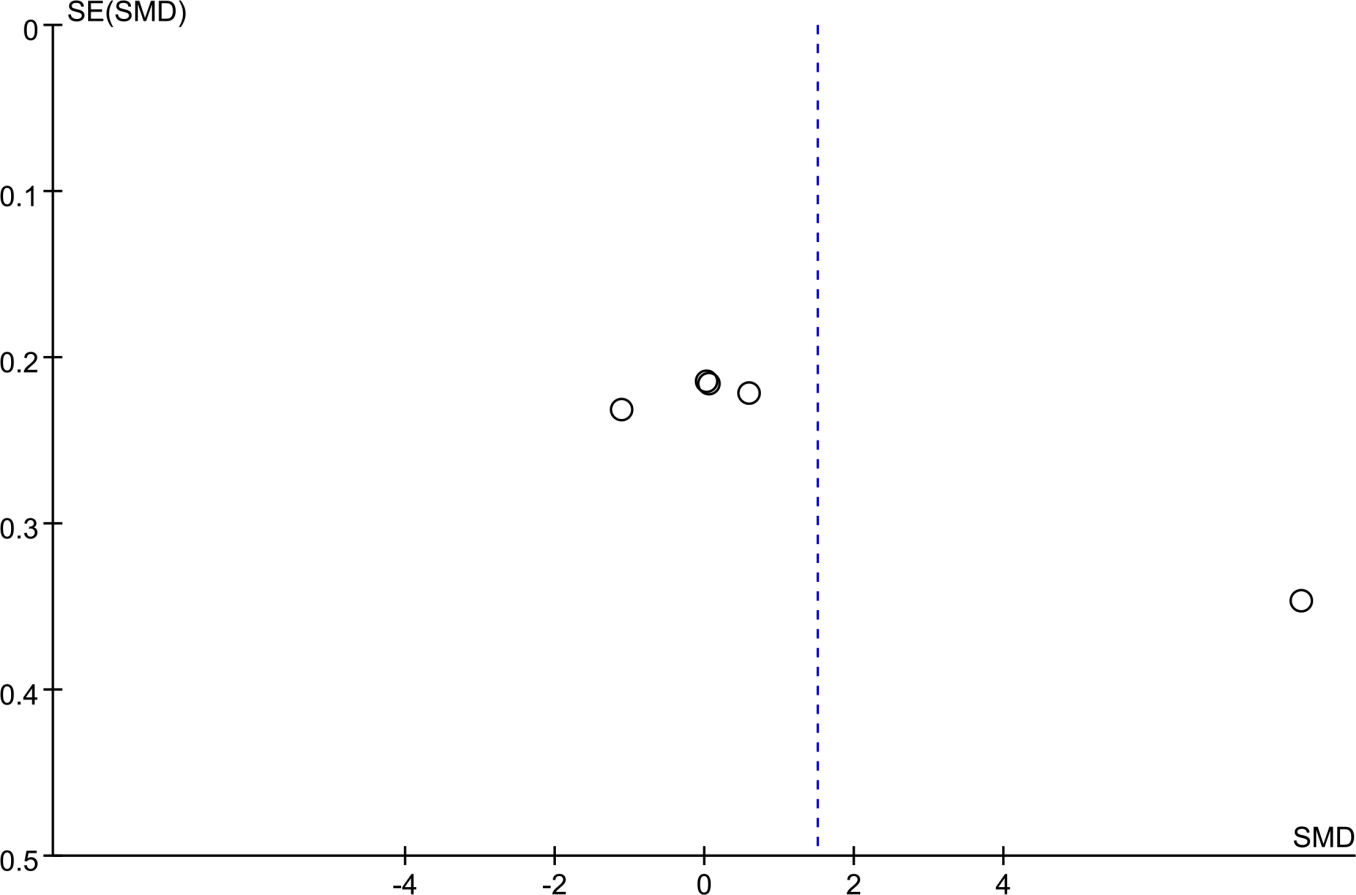
TCMSS funnel plot.

Funnel plots for PCV (**Figure 4**) and the total score of TCMSS (**Figure 5**) were asymmetrical, indicating potential publication bias. The detailed results are provided in **Supplementary Figures S3**, **S4**. Egger's test confirmed significant bias for these outcomes (p = 0.028, p = 0.023). For other outcomes, encompassing 2h PG (p = 0.583), BMI (p = 0.598), FBG (p = 0.349), glycated HbA1c (p = 0.112), HDL (p = 0.097), Homa-B (p = 0.190), ISI (p = 0.348), LDL(p =0.219), Plasma Viscosity (p = 0.113), TC(p = 0.161),TG (p = 0.211), and Whole Blood Viscosity (p = 0.495), no publication bias was detected (**Supplementary Figures S5**, **S6**).

### 3.7 Sensitivity analysis

**Figure 6:**
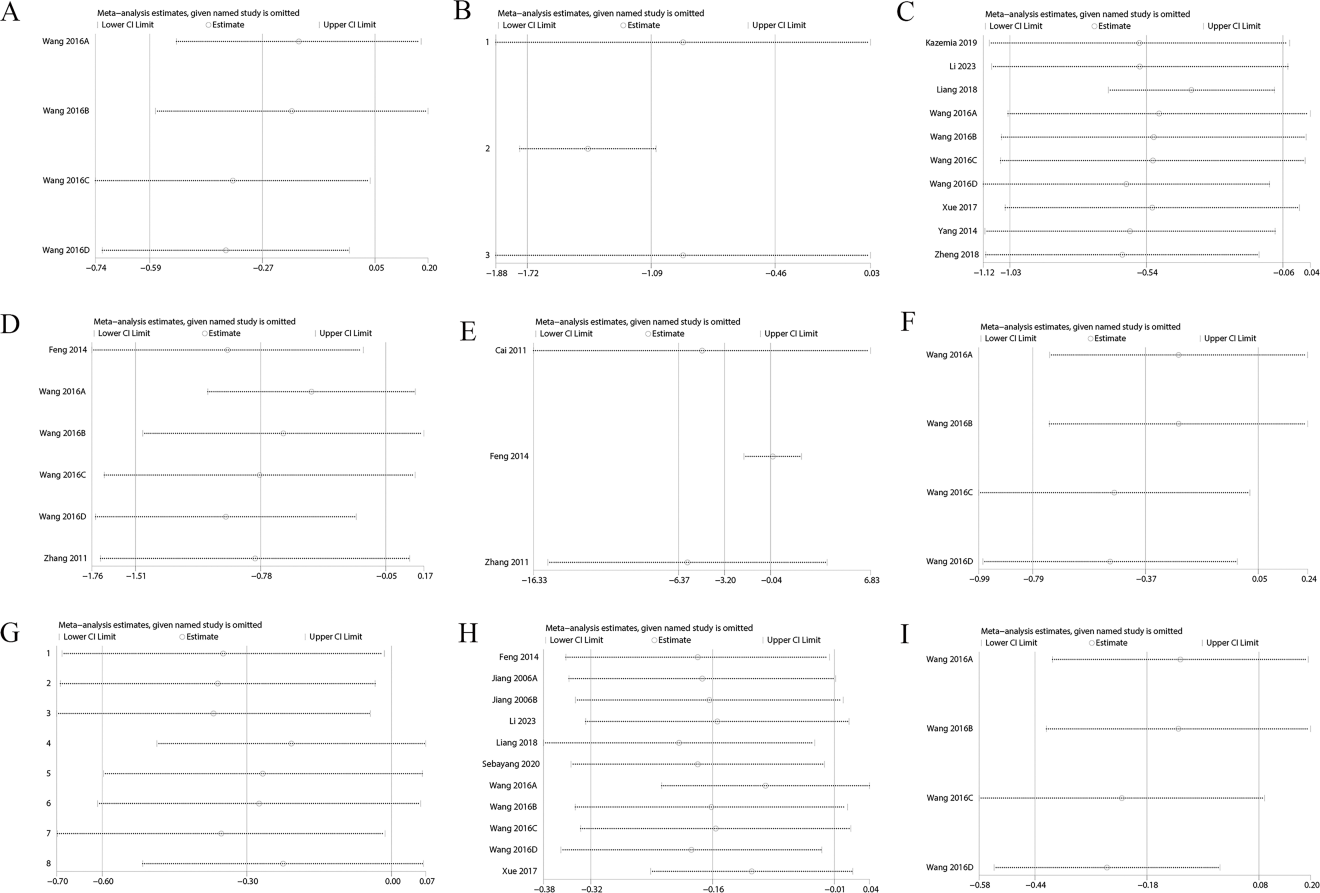
**(A)** FIB; **(B)** FINS; **(C)** HbA1c; **(D)** Homa-B; **(E)** ISI; **(F)** PCV; **(G)** TC; **(H)** TG; **(I)** Whole Blood Viscosity.

**Figure 7:**
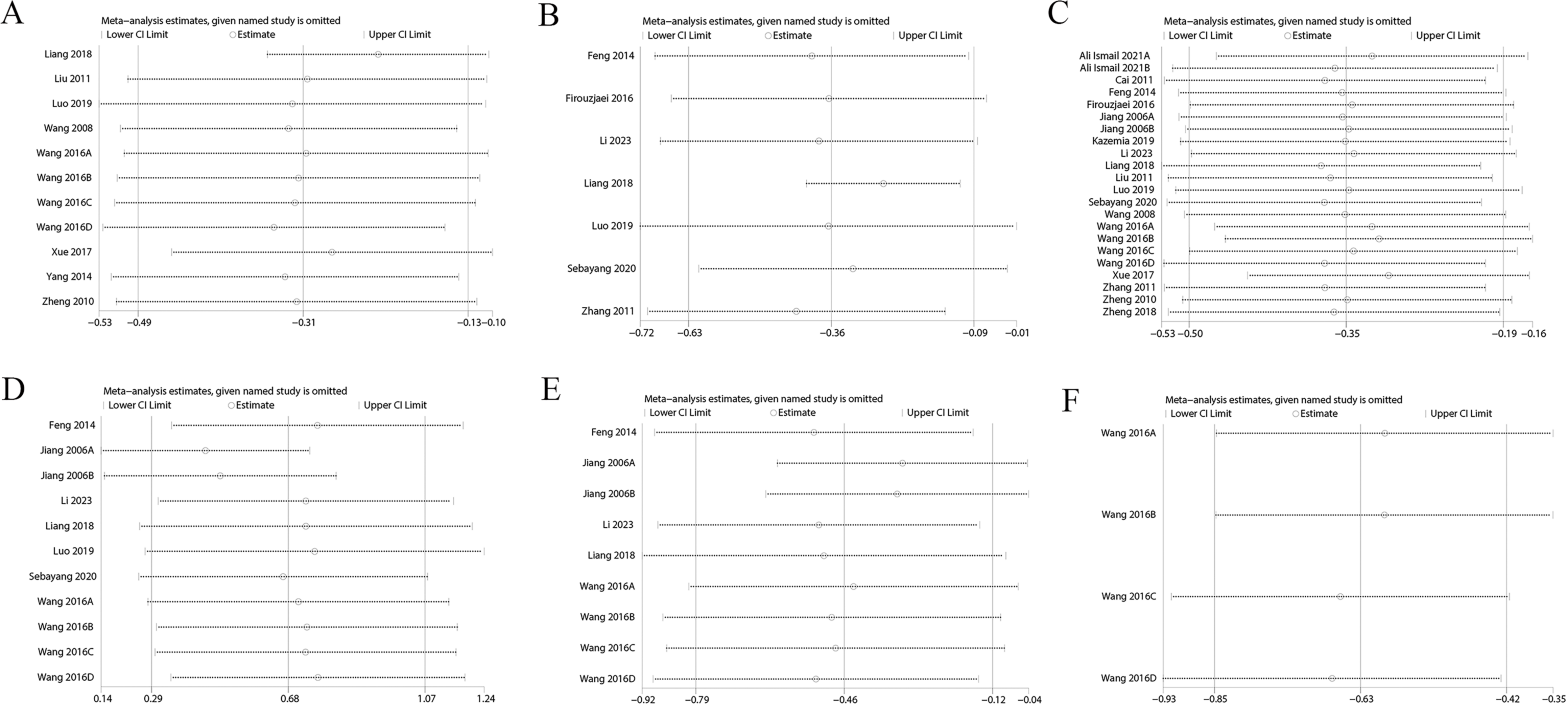
**(A)** 2h PG; **(B)** BMI; **(C)** FBG; **(D)** HDL; **(E)** LDL; **(F)** Plasma Viscosity.

Sensitivity analysis showed variations in effect sizes for FIB, FINS, HbA1c, Homa-B, ISI, PCV, TC, TG, and whole blood viscosity, indicating that some studies imposed a disproportionate influence on the analysis results. Hence, the above results were not robust (**Figure 6**, **Supplementary Figure S7**). However, outcomes such as 2h PG, BMI, FBG, HDL, LDL, and plasma viscosity exhibited stable effect sizes, confirming their robustness (**Figure 7**, **Supplementary Figure S8**).

### 3.8 Subgroup analysis

Subgroup analysis of FBG in T2DM was implemented by patient population, treatment duration, region, and mean age of patients. Acupuncture did not significantly affect FBG in studies on T2DM patients with obesity, prediabetes, or impaired glucose tolerance, or those conducted outside of China. Thread-embedding acupuncture and acupuncture plus drugs had no significant effects. However, significant improvements in FBG were observed in other subgroups. The primary source of heterogeneity was related to simple T2DM, treatment durations of shorter than 3 months, and acupuncture methods such as traditional acupuncture and electroacupuncture.

Subgroup analysis for 2h PG was executed based on the same factors. Acupuncture showed no significant effects in studies on simple T2DM, impaired glucose tolerance, treatment durations of less than 3 months, or patients aged 50 years or older. Significant improvements were observed in other subgroups, with no major sources of heterogeneity identified.

Subgroup analysis for TG, a secondary outcome, was performed by patient population, treatment duration, region, and mean age. The results were unstable. Acupuncture ameliorated TG only in studies involving T2DM with complications, treatment durations of more than 3 months, and those conducted in China Other subgroups showed no significant effects. Detailed results are provided in **Supplementary Table S4**.

In the subgroup analysis of the efficacy of acupuncture methods, the results may not reliable due to the limited number of included studies. In the future, more multi-center studies with large sample sizes are needed, and different acupuncture treatment methods should be compared to determine the best acupuncture treatment method.

## 4 Discussion

T2DM, a metabolic condition influenced by genetic, autoimmune, and environmental factors, represents over 95% of diabetes cases. It is estimated that over 500 million individuals will be afflicted by 2045 (29), contributing to a heavy social and economic burden. This highlights the pressing need for effective management strategies to address its societal and economic burdens. Consequently, early diagnosis, treatment, and management are crucial for relieving symptoms, enhancing the quality of life, and improving prognosis in type 2 diabetes patients. TCM, particularly acupuncture, which emphasizes holistic regulation, has garnered increasing interest for its potential to manage T2DM and its complications (30). Previous research by Qin Fulan et al. (31) demonstrated significant reductions in FBG with acupuncture, while Robby Gunawan Sebayang et al. (21) found that acupuncture had limited effects in patients with obesity. These discrepancies may stem from variations in syndrome types and acupuncture points selected. Consequently, it is imperative to systematically ascertain the effects of acupuncture on blood glucose control in T2DM.

The current study disclosures that acupuncture can considerably ameliorate a variety of clinical outcomes in T2DM, encompassing glycated hemoglobin, 2h PG tolerance, FBG, fasting insulin, Homa-IR, Homa-B, TG, BMI, LDL, HDL, WHR, plasma viscosity, and nerve conduction velocities, with no publication bias. Sensitivity analysis reveals that outcomes such as 2h PG tolerance, FBG, BMI, and WHR show stable results. Nonetheless, the results for glycated hemoglobin, FINS, Homa-IR, and TG are unstable, indicating limited evidence quality for these measures. Consistent with the study by Zhang et al. (32), acupuncture was found to be safe, effective, and fast-acting. Li SQ et al. uncover (7) a modest but significant effect of acupuncture on FBG and insulin resistance. The inclusion of larger, more recent studies provides updated evidence on acupuncture's efficacy in managing T2DM. The effects of acupuncture on HbA1c, 2h PG, and FINS remain elusive. These findings align with those of the current study. On the contrary, the current study unveils that acupuncture treatment can improve HbA1c, 2h PG, FINS, ISI, TG, BMI, LDL, HDL, and WHR. Furthermore, the articles included in this research were of higher quality, published more recently, and had larger sample sizes, offering more reliable, the most up-to-date and comprehensive evidence for acupuncture in the treatment of T2DM.

Subgroup analysis by patient populations, treatment durations, regions, and mean age indicated that acupuncture was less effective for FBG and 2h PG in patients with obesity, prediabetes, or impaired glucose tolerance, possibly because compared to simple T2DM, obesity-related fat tissue secretes adipokines that affect glucose and lipid metabolism, and leptin deficiency can lead to obesity and insulin resistance (33, 34). Obese patients have elevated levels of inflammatory factors such as interleukin-6, which can induce insulin resistance (35). Another study (36) unravels that T2DM patients with overweight or obesity have lower levels of myonectin compared to T2DM patients, and myonectin plays a role in fat tissue and regulates glucose and lipid metabolism. Myonectin levels are negatively linked to insulin resistance and BMI in T2DM patients, and they are an independent factor affecting insulin resistance and BMI. A decrease in myonectin levels can contribute to the onset of overweight and obesity by affecting fat metabolism. In the studies on FBG, heterogeneity was likely associated with studies involving simple T2DM and treatment durations of less than 3 months. Future research should compare these indicators, enroll different patients with various underlying diseases, use different acupuncture methods, focus on multiple treatment durations to reduce the impact of heterogeneity on results.

For prediabetic patients, the lack of significant differences may be due to less pronounced blood glucose fluctuations and insulin resistance. In terms of research regions, acupuncture originated in China and has been utilized and validated in clinical practice for over two thousand years (37). This may explain the higher number of studies in China compared to other regions, where fewer studies may yield false-negative results. Moreover, acupuncture relies heavily on the practitioner's skill and experience, and Chinese practitioners may have more advanced techniques, resulting in more effective outcomes in Chinese studies. In the subgroup analysis of 2h PG, although blood glucose control was achieved in the short term for patients with simple T2DM and impaired glucose tolerance, there was no considerable difference in long-term blood glucose stability. The specific mechanisms remain to be further explored. Acupuncture can regulate the autonomic nervous system to adjust insulin secretion and glucose regulation, and may also influence the endocrine system. A short treatment duration may not allow the body to achieve a stable equilibrium. In patients aged 50 years and older, acupuncture may be less effective due to the aging of their nervous and endocrine systems.

Acupuncture is a safe and effective treatment, and recent research on acupuncture for T2DM has shown promising results. Acupuncture has been demonstrated to reduce insulin and leptin levels in T2DM patients, increase serum adiponectin levels, alleviate insulin resistance, and regulate glucose and lipid metabolism. Acupuncture also affects the neural ultrastructure and neurophysiology (15, 38), and can change the density and morphology of nerve fibers. Through light and electron microscopy, it has been observed that acupuncture increases the density of nerve fibers and changes the morphology of nerve cells. These changes may be related to the bioactive substances produced by nerve cells after acupuncture stimulation. Additionally, through EEG and neurophysiological recordings, acupuncture can alter electrical signal transmission between neurons, regulating the frequency and amplitude of brain waves (39). These changes may be linked to the effects of acupuncture on neurotransmitters. Acupuncture can also influence T2DM by regulating the endocrine system. Research shows that acupuncture can affect the hypothalamic-pituitary-adrenal axis and the hypothalamic-pituitary-thyroid axis, promoting hormonal balance and further controlling blood glucose levels (40).

The most commonly used acupuncture point in this study was ST36. Research has shown (41) that acupuncture at ST36 can ameliorate insulin sensitivity and the morphology of pancreatic β-cells. Acupuncture for T2DM primarily targets the foot yangming stomach meridian, and foot taiyang bladder meridian, along with RN12, RN4, and other Renmai acupoints. In TCM, diabetes is classified as "Xiao Ke," involving the heart, lung, spleen, stomach, liver, and kidneys. The symptoms of T2DM can be summarized as excessive eating, drinking and urinating. In TCM theory, the lung can transport water upward and outward to the whole body. The spleen and stomach play a pivotal role in transporting nutrients and water to the whole body. If the spleen and stomach are damaged, weight loss, excessive eating and sweet urine will occur. The main function of the liver is to dredge the qi mechanism. The kidney has the physiological function of presiding over and regulating water metabolism. The liver and kidney can also help the spleen and stomach transport nutrients and improve the normal metabolism of sugar and fat. If the liver and kidneys are damaged, symptoms such as dry mouth, sore waist and knees, and frequent urination will occur. ST36 is the He point of the Stomach Meridian of Foot Yangming. Modern research has found (42) that the sensory fibers of ST36 and the afferent nerves of the stomach in newborn rats converge and overlap in the dorsal root ganglia of the T12-L7 segment. Acupuncture signals are transmitted from ST36 through somatic nerves, mainly certain vascular wall nerve plexuses, and the common peroneal nerve to the thoracic and lumbosacral segmental nerve roots, and finally exchange and transmit signals with gastrointestinal afferent nerves. This may be one of the specific effect mechanisms of ST36 (43). Back Shu acupoints belong to the foot-sun bladder meridian, including the places where the meridian qi of the five internal organs and six bowels is infused on the back. It is a general term for some specific acupoints such as BL13, BL23, and BL28, where the qi of the internal organs is infused on the back. They are located near the corresponding internal organs. Because of the mutual communication of qi and blood and the internal and external relationship between the internal organs, stimulating the bladder meridian can regulate the function of the corresponding internal organs. The bladder is the place where body fluids are stored, and it is mainly responsible for urination after the qi is transformed by the kidney. The kidney controls the water of the whole body, and the water metabolism of the whole body needs to be regulated by the kidney. If the kidney is normal, the water distribution of the whole body is normal (44). Animal experiments have unraveled that acupuncture at RN12 can heighten the expression levels of corticotropin-releasing hormone (CRH) and c-fos protein in the hypothalamus, as well as the levels of peripheral insulin and β-endorphin, exerting a pivotal role in managing blood sugar (45). RN4 is a small intestine acupoint located at the intersection of the Ren Meridian and the three Yin meridians of the foot, and is linked to life energy. SP36 is located at the intersection of the three Yin meridians of the foot. Chronic T2DM is mostly caused by a deficiency of both Qi and Yin, accompanied by dry heat. Usually, RN4 and SP36 acupuncture points are activated to improve the Qi and Yin of the liver, spleen, and kidney (46). CV12 belongs to the Ren meridian point and can regulate the digestion, absorption, and secretion functions of the stomach (47). It has been demonstrated that electroacupuncture at CV12 can significantly increase peripheral β-endorphin levels, promote insulin secretion and release, and effectively relieve hyperglycemia; at the same time, electroacupuncture can change the endocrine function of the hypothalamus, correct blood sugar disorders by affecting the hypothalamic-pituitary-adrenal axis, and thus regulate abnormal endocrine metabolism (48).

This study incorporates 21 RCTs on acupuncture for T2DM. This is the latest meta-analysis in this field, and all included studies are high-quality RCTs. Furthermore, subgroup analysis was implemented, aiming to identify differences in efficacy and sources of heterogeneity across different populations. The results confirm that acupuncture is effective and safe for individuals with T2DM. By exploring the sources of heterogeneity through multidimensional subgroup analysis, the study provides an evidence-based basis for further clinical research. Nonetheless, this study has some limitations. Owing to variations in acupuncture points, techniques, and the pathological features of complications among the included studies, high heterogeneity was noted for some outcomes. Through subgroup analysis, the sources of the heterogeneity were identified and explained. Despite some studies with no clear blinding and allocation procedures, this limitation did not significantly affect our results. Most studies were carried out in China, which may have led to publication and regional selection biases. Further high-quality experiments are warranted to enhance the level of evidence. Sensitivity analysis unraveled unstable results.

## 5 Conclusion

This study demonstrates that acupuncture, as an auxiliary treatment, can relieve the clinical symptoms of T2DM to a certain extent, such as improving ISI, HbA1c, 2h PG, FBG, FINS, Homa-IR, Homa-B, TG, BMI, LDL, HDL, WHR, plasma viscosity, bilateral median nerve motor conduction velocity, and bilateral common peroneal nerve motor conduction velocity. These findings uncover that acupuncture appears to be a safe and potentially effective treatment strategy for T2DM. However, given the potential heterogeneity, publication bias, small sample sizes, and regional selection bias, further large-scale, multicenter RCTs are warranted to corroborate the clinical benefits of acupuncture for individuals with T2DM and explore possible influencing factors.

## Data Availability

The original contributions presented in the study are included in the article/**Supplementary Material**. Further inquiries can be directed to the corresponding author.

## Supplementary material

Supplementary Table 1 Summary Table of Clinical Studies on Acupuncture Treatment for Type 2 Diabetes Mellitus

Supplementary Table 2 Standard Terminologies and Functions of Acupuncture Points

Supplementary Table 3 Distribution and Frequency of Acupuncture Points in Meridians Ranking Table

Supplementary Table 4 Subgroup Analysis Results of the Impact of Acupuncture

Supplementary Figure 1
**(A)** Forest plot FINS; **(B)** Forest plot Homa-IR; **(C)** Forest plot Homa-B; **(D)** Forest plot TG; **(E)** Forest plot BMI; **(F)** Forest plot LDL; **(G)** Forest plot HDL; **(H)** Forest plot WHR; **(I)** Forest plot Plasma Viscosity.

Supplementary Figure 2
**(A)** Forest plot Bilateral median nerve motor conduction velocity; **(B)** Forest plot Bilateral common peroneal nerve motor conduction velocity; **(C)** Forest plot TC; **(D)** Forest plot body weight; **(E)** Forest plot body fat percentage; **(F)** Forest plot PCV; **(G)** Forest plot whole blood viscosity; **(H)** Forest plot FIB; **(I)** Forest plot Scr.

Supplementary Figure 3
**(A)** Funnel plot ISI; **(B)** Funnel plot 2h PG; **(C)** Funnel plot Bilateral common peroneal nerve motor conduction velocity; **(D)** Funnel plot Bilateral median nerve motor conduction velocity; **(E)** Funnel plot BMI; **(F)** Funnel plot body fat percentage; **(G)** Funnel plot body weight; **(H)** Funnel plot FBG; **(I)** Funnel plot FIB; **(J)** Funnel plot FINS; **(K)** Funnel plot HbA1c; **(L)** Funnel plot HDL.

Supplementary Figure 4
**(A)** Funnel plot Homa-B; **(B)** Funnel plot Homa-IR; **(C)** Funnel plot Insulin levels; **(D)** Funnel plot LDL; **(E)** Funnel plot PCV; **(F)** Funnel plot Plasma Viscosity; **(G)** Funnel plot Scr; **(H)** Funnel plot TC; **(I)** Funnel plot TCMSS; **(J)** Funnel plot TG; **(K)** Funnel plot whole blood viscosity; **(L)** Funnel plot WHR.

Supplementary Figure 5
**(A)** 2h PG; **(B)** BMI; **(C)** FBG; **(D)** glycated HbA1c; **(E)** HDL; **(F)** Homa-B; **(G)** ISI; **(H)** LDL; **(I)** PCV;

Supplementary Figure 6
**(A)** TC; **(B)** TG; **(C)** Whole Blood Viscosity; **(D)** Plasma Viscosity; **(E)** TCMSS; **(F)** FIB

Supplementary Figure 7
**(A)** FIB; **(B)** FINS; **(C)** HbA1c; **(D)** Homa-B; **(E)** ISI; **(F)** PCV; **(G)** TC; **(H)** TG; **(I)** Whole Blood Viscosity

Supplementary Figure 8
**(A)** 2h PG; **(B)** BMI; **(C)** FBG; **(D)** HDL; **(E)** LDL; **(F)** Plasma Viscosity; **(G)** TCMSS
