# Supplementary material for: The effect of acupuncture on blood glucose control in patients with type 2 diabetes: a systematic review and meta-analysis of randomized controlled trials
Source: Front Endocrinol (Lausanne). 2025 Jun 11;16:1596062. doi: 10.3389/fendo.2025.1596062 (PMC12187737; doi:10.3389/fendo.2025.1596062)
Supplement: Supplementary Table 2 — Standard Terminologies and Functions of Acupuncture Points [file Table2.docx]

**Table S2** Standard Terminologies and Functions of Acupuncture Points

| Pinyin | standard terminologies | Location | Function |
| --- | --- | --- | --- |
| Yishu | EX-B3 | It is located on the back, 0.5 cun lateral to the lower border of the spinous process of the 8th thoracic vertebra. | It is mainly used in the treatment of diabetes, abdominal pain, and poor digestion. It can help regulate qi movement in the abdomen, improve digestive function, and may have certain regulatory effects on blood - sugar levels in some cases. |
| Feishu | BL13 | The Feishu acupoint (BL13) is located on the back, 1.5 cun lateral to the lower border of the spinous process of the third thoracic vertebra. | It can be used for respiratory diseases. For example, it is beneficial for relieving coughs, whether they are caused by colds, bronchitis or other respiratory problems.  It also helps in improving the function of the lungs, such as enhancing the lung's qi (vital energy in traditional Chinese medicine). It may be helpful for some people with shortness of breath or weak lung function. |
| Pishu | BL20 | The Pishu acupoint is located on the back, at the level of the 11th thoracic vertebra, 1.5 cun (a unit of measurement in traditional Chinese medicine) lateral to the posterior midline. | It can regulate the function of the spleen. For example, it may help improve the digestion and absorption function of the spleen when it is in a disordered state, such as alleviating symptoms like abdominal distension and poor appetite.  It also has a certain role in strengthening the body's qi (vital energy). It can be beneficial for enhancing the overall physical condition and resistance when the body is weak due to spleen deficiency. |
| Weishu | BL21 | It is located on the back, 1.5 cun lateral to the lower border of the spinous process of the 12th thoracic vertebra. | It can regulate the function of the stomach. For example, it can be used to relieve stomach pain, bloating, and poor digestion.  It also has a certain role in strengthening the spleen and stomach qi. |
| Shenshu | BL23 | Shenshu is located on the lower back, 1.5 cun lateral to the lower border of the spinous process of the second lumbar vertebra. (Here, "cun" is a traditional Chinese measurement unit in acupuncture.) | It can tonify the kidney qi. For example, it is helpful for some symptoms related to kidney deficiency such as weakness in the lower back and knees.  It has a role in regulating water metabolism in the body.  It can also improve the function of the reproductive system to a certain extent. |
| Zusanli (stomach 36) | ST36 | It is located on the anterior aspect of the lower leg, 3 cun (a traditional Chinese measurement unit) below Dubi (ST35), one finger - breadth (middle finger) from the anterior crest of the tibia. | It can regulate the function of the stomach and intestines. For example, it can relieve abdominal pain, diarrhea, and constipation, strengthen the body's immunity, and regularly stimulate this acupoint, which may help the body resist diseases better.  Improve overall vitality. It is often used in traditional Chinese medicine to relieve fatigue and boost energy. |
| Sanyinjiao | SP6 | Sanyinjiao is located on the medial side of the lower leg, about 3 cun (a unit of measurement in traditional Chinese medicine, approximately the width of four fingers) above the tip of the medial malleolus, posterior to the medial border of the tibia. | For the gynecological aspect, it can regulate menstruation, relieve dysmenorrhea, and is beneficial for some female reproductive system disorders.  In terms of general health, it can help strengthen the spleen and stomach, improve digestion, and also has certain effects on relieving insomnia and anxiety by regulating the qi and blood in the body. |
| Quchi | LI11 | It is located at the lateral end of the transverse cubital crease, midway between Chize (LU5) and the lateral epicondyle of the humerus. | It can be used for relieving exterior syndromes such as fever, headache, and cough.  It is also effective in treating diseases of the upper limbs, like elbow pain and paralysis of the upper extremities.  Moreover, it has certain functions in regulating qi and blood, and improving the function of the digestive system. |
| Hegu (large intestine 4) | LI4 | Hegu is located on the dorsum of the hand, between the first and second metacarpal bones, approximately at the midpoint of the second metacarpal bone. | It can relieve pain. For example, it is often used for headaches, toothaches, and other pain symptoms.  It has certain regulatory effects on the face, such as for relieving facial paralysis symptoms to a certain extent.  It can also help with some febrile diseases, playing a role in reducing fever in traditional Chinese medicine theory. |
| Fenglong | ST40 | 1. It is located on the anterior aspect of the leg. 2. Specifically, it is about 8 cun above the tip of the external malleolus, and 1 cun lateral to the anterior crest of the tibia. | **1.Phlegm - resolving**  It is very effective in resolving phlegm. For example, in traditional Chinese medicine, it can be used to treat excessive phlegm in the body, such as cough with copious phlegm.  **2.Stomach - regulating**  It can regulate the function of the stomach. If a person has problems like indigestion, nausea, or stomach distension, stimulating Fenglong point may help relieve these symptoms. |
| Yinlingquan (spleen 9) | SP9 | It is located on the medial side of the lower leg, in the depression posterior and inferior to the medial condyle of the tibia. | Diuretic effect: It can help regulate water metabolism in the body and promote urination.  Strengthening the spleen and stomach: It has a certain role in improving the function of the spleen and stomach, for example, relieving symptoms such as abdominal distension and poor digestion. |
| Zhongwan | CV12/RN12 | It is located on the anterior midline of the upper abdomen.  Specifically, it is 4 cun above the umbilicus. | 1.Regulating the function of the stomach  It can help with problems such as stomachache. For example, if a person has a dull pain in the stomach due to improper diet, stimulation of Zhongwan acupoint may relieve the pain.  2.Improving digestion  By promoting the movement of Qi (vital energy in traditional Chinese medicine) in the digestive system, it can enhance the digestion process. This can be beneficial for people with indigestion, bloating and other symptoms.  3.Harmonizing the middle - Jiao  In traditional Chinese medicine theory, the middle - Jiao is related to the functions of the stomach and spleen. Zhongwan acupoint can help to balance the functions of these organs and improve the overall condition of the middle - Jiao. |
| Ganshu | BL18 | It is located on the back, 9 - cun lateral to the lower border of the spinous process of the ninth thoracic vertebra. | 1. For liver diseases   It can be used to regulate liver qi, which is helpful for relieving symptoms such as liver depression and qi stagnation.   1. Digestive function   It has an impact on the digestive system. It can help improve digestion, relieve abdominal distension and other problems related to the disorder of liver - related digestion.   1. Eye problems   It is also related to eye health. It may help in some eye disorders because in traditional Chinese medicine, the liver is related to the eyes. |
| Ququan | LR8 | Ququan acupoint is located on the medial side of the knee in the human body. When the knee is flexed, it is at the medial end of the transverse striation on the medial surface of the knee joint, the posterior border of the medial condyle of the femur, and the anterior border of the depression at the end of the semitendinosus and semimembranosus muscles. | It can regulatemenstruation and arresting leucorrhea, clear heat and promoting diuresis, sooth the liver and regulating qi, activate blood and relieving pain, dredge and regulate the lower - jiao. |
| Xiaxi | GB43 | Xiáxī is located on the foot, in the depression distal to the 4th and 5th metatarsal bones, on the lateral side of the tendon of the extensor digitorum longus muscle. | It can help relieve headache, vertigo.  It has certain effects on relieving tinnitus, deafness.  It can also be beneficial for alleviating pain in the hypochondriac region and regulating qi in the gallbladder meridian. |
| Taichong | LR3 | It is located on the dorsum of the foot, in the depression distal to the junction of the first and second metatarsal bones. | 1.Liver - related functions  It can soothe the liver and regulate qi. For example, it is helpful for relieving symptoms such as irritability and depression which are often related to liver - qi stagnation.  2.Blood - related functions  It can promote blood circulation. It may be used in some cases to help with menstrual disorders by regulating the blood flow in the body. |
| Guanyuan | CV4/RN4 | Guanyuan is located on the anterior midline of the lower abdomen, 3 cun below the umbilicus. | Tonifying qi: It can strengthen the body's qi (vital energy), which is beneficial for people with qi deficiency.  Nourishing the kidney: It has a positive effect on the kidney, for example, it can help improve some symptoms related to kidney deficiency such as fatigue, lumbago and so on.  Regulating menstruation: For women, it can play a role in regulating the menstrual cycle and relieving menstrual discomfort. |
| Zhigou | TE6 | On the forearm, 3 cun above the transverse crease of the wrist, between the radius and ulna. | It can regulate qi and relieve constipation.  It is also used for treating pain in the chest and hypochondriac region, and some symptoms related to qi stagnation in the Triple Energizer. |
| Zhaohai | KI6 | Zhaohai is located on the medial side of the foot, about 1 cun directly below the tip of the medial malleolus, in the depression between the medial malleolus and the calcaneus. | It can be used to treat insomnia. For example, acupuncture at this point may help regulate the body's internal rhythm and improve sleep quality.  It is beneficial for throat problems. Such as relieving sore throat.  It may also play a role in regulating the kidney - related functions in traditional Chinese medicine theory. |
| Chongyang | ST42 | It is located on the dorsum of the foot, in the depression distal to the junction of the second and third metatarsal bones and cuneiform bones. | It can be used to regulate the stomach qi. For example, it can help relieve symptoms such as stomachache, abdominal distension which are related to disorder of stomach qi.  It also has a certain effect on improving digestion. By promoting the normal movement of qi in the stomach and intestines, it can enhance the digestive function. |
| Neiting | ST44 | It is on the dorsum of the foot, in the depression distal to the junction of the second and third metatarsal bones and the second and third cuneiform bones, at the lateral side of the tendon of the extensor digitorum longus muscle. | It can be used for treating some disorders in the head, such as headache, toothache, etc.  It also has certain effects on treating problems in the face area like facial swelling, epistaxis.  Helps with gastrointestinal problems to a certain extent, for example, it can be beneficial for stomach pain and abdominal distension. |
| Tianshu | ST25 | Tian Shu is located on the abdomen, 2 cun lateral to the center of the umbilicus. | It can regulate the qi of the intestines and stomach. For example, it is often used to treat abdominal pain, diarrhea, constipation and other symptoms related to the disorder of the intestines and stomach qi. It also has a certain effect on strengthening the spleen and stomach function. |
| Yanglingquan | GB34 | It is in the depression anterior and inferior to the head of the fibula. | It can relieve pain in the lower extremities, such as pain in the knees and ankles.  It has a certain regulatory effect on the liver and gallbladder, which can be used to treat some liver and gallbladder disorders, like jaundice in traditional Chinese medicine theory.  It also can dredge the meridians and collaterals, which is beneficial for improving the circulation of qi and blood in the body. |
| Dazhui | GV14/DU14 | It is located in the posterior midline of the neck, below the spinous process of the 7th cervical vertebra. | It can help relieve fever. For example, in cases of common cold with fever, stimulating Dazhui may play a role in reducing body temperature.  It is beneficial for relieving pain in the neck and shoulders. People with neck and shoulder pain due to long - term sitting or incorrect posture may get some relief by proper stimulation of this acupoint.  It can also help improve the function of the immune system to a certain extent, enhancing the body's resistance to diseases. |
| Qihai | CV6 | On the anterior midline of the lower abdomen, 1.5 cun below the umbilicus. | It can regulate qi (vital energy in traditional Chinese medicine), strengthen the spleen and stomach, warm the lower jiao (the lower part of the body in TCM concept including the kidney, bladder, intestines etc.), and has certain effects on relieving abdominal pain, diarrhea, and regulating qi - related disorders in the body. |
| Shaoshang | LU11 | On the thumb, at the radial side of the thumb nail, 0.1 cun from the corner of the nail. | It can be used for relieving sore throat, as it has a certain effect on reducing heat in the lung meridian.  It can also help with some symptoms related to febrile diseases, such as fever, cough and so on. |
| Jinjin | EX-HN12 | on the posterior aspect of the tongue,the left and right sides of the frenulum linguage. | It can clear heat and promot fluid production |
| Yuye | EX-HN12 | on the posterior aspect of the tongue, at the veins on both sides of the frenulum of the tongue. | It can clear heat and purge fire,promoting fluid production and relieving thirst |
| Chengjiang | RN24 | In the depression of the centre of the mentolabial groove. | It can regulate salivation, relieve facial pain, treat orofacial disorders |
| Yangchi | SJ4 | On the dorsum of the wrist, in the depression between the tendons of extensor digitorum communis, extensor digiti minimi and the distal end of the radius. | It can regulate qi, promote the circulation of qi - blood, dispel cold - dampness, benefit the eyes |
| Daheng | SP15 | On the abdomen, 4 cun lateral to the centre of the umbilicus. | It can regulate the function of the spleen and stomach, and promote qi movement in the intestines |
| Taixi | KI3 | In the depression between the tip of the medial malleolus and the Achilles tendon. | It can nourish Kidney - Yin, strengthen the kidneys. regulate water metabolism, relieve pain in the lower limbs and back. |
| Rangu | KI2 | on the medial side of the foot, in the depression distal to the navicular tuberosity, anterior to the medial malleolus. | It can regulate blood sugar, nourish yin and reducing fire, benefit the kidneys and promote diuresis |
| Waiguan | SJ5 | On the line joining Yangchi and the tip of elbow, 2 cun above the dorsocarpal transverse crease, between the ulna and radius. | It can dredge the channels and collaterals, dispel pathogenic factors, and regulate the triple energizer. |
| Xuehai | SP10 | 2 cun above the upper border of the medial patella;or the acupoint is where the tip of the thumb when doctor puts his right palm on the left knee of patient and with the centre of the palm pointing to the centre of patella of patient and the patient flexes his knee joint and makes it to be a right angle. | It can regulate menstruation, nourish and regulate blood, and treat skin diseases. |
| Chengshan | BL57 | In the depression below the belly of the gastrocnemius m. when stretching the leg or lifting the heel. | It can relieve muscle spasms, dredge the meridians, promote qi - blood circulation, and treat hemorrhoids |
| Taibai | SP3 | Prosteroinferior to the the head of the 1st metatarsal bone,and on the dorso-ventral boundary. | It can regulate the spleen and stomach, and tonify qi and blood. |
| Bafeng | EX-LE10 | On the dorsum of the foot, between the toes, proximal to the margins of the webs, 0.5 cun proximal to the border of the web, four on each foot. | It can relieve foot pain, dispel pathogenic wind, and clear heat and reduce swelling. |
| Xiyan | EX-LE5 | lateral xiyan:on the anterior aspect of the knee, in the depression lateral to the patellar ligament.  medial xiyan:on the anterior aspect of the knee, in the depression medial to the patellar ligament. | It can relieveknee pain, strengthen the knee joint, and treat disorders related to the knee. |
| Shuifen | RN9 | on the anterior midline of the abdomen, 1 cun above the umbilicus. | It can regulate water metabolism, and treat abdominal disorders. |
| Shuidao | ST28 | On the lower abdomen, 3 cun lateral to the centre of the umbilicus. | It can regulate water passages, treat genitourinary disorders, and alleviate abdominal pain |
| Jianyu | LI15 | In the depression between the acromial extremity of the clavicle and the great tuberosity of humerus;or when the arm is in full abduction,the acupoint is in the depression at the anterior border of the acromioclavicular joint,and superior to the shoulder joint. | It can relieve shoulder pain, enhance shoulder joint mobility, and dispel pathogenic factors in the shoulder. |
| Jianliao | SJ14 | In the depression poteroinferior to the acromion when arm is abducted. | It can relieve shoulder pain, enhance shoulder joint mobility, and dispel pathogenic factors in the shoulder. |
| Huantiao | GB30 | At the junciton of the medial 2/3 and lateral 1/3 of the line joining the prominence of the great trochanter and the sacral histus. | It can relieve hip and leg pain, improve lower - limb mobility, and dispel pathogenic wind and cold. |
| Jiexi | ST41 | At the midpoint of the transverse crease of the junction between the dorsum of foot and leg,and between the tendons of long extensor m. of great toe and long extensor m. of toes. | It can relieve ankle pain, dredge the meridians, promote qi - blood circulation, and regulate the function of the stomach and intestines. |
| Xingjian | LR2 | On the dorsum of the foot, between the first and second toes, proximal to the margin of the web, at the junction of the red and white skin. | It can regulate liver - qi, reduce liver - fire, and promote blood circulation. |
| Neiguan | PC6 | On the line joining Daling and Quze , between the tendons of palmaris longus and flexor carpi radialis, 2 cun above the transverse crease of the wrist. | It can regulate the heart and chest, calm the wind and regulate the stomach. |
| Geshu | BL17 | 1.5 cun lateral to the depression below the spinous process of the 7^th^ thoracic vertebra. | It can nourish blood, activate blood circulation, remove blood stasis, and regulate the function of the diaphragm. |
| Dacha |  | At the red and white meat midpoint between 1st metacarpophalangeal joint and 2nd metacarpophalangeal joint. | It can regulate qi - balance and enhance water - metabolism. |
| Huozhu |  | In the depression anterior to the junction of 1^st^ and the 2^nd^ metatarsal bones. | It can treat liver and gallbladder disorders, calm the mind, relieve stress, and regulate menstruation. |
| Huolian |  | Prosteroinferior to the head of the 1^st^ metatarsal bone, and on the dorso-ventral boundary. | It can treat digestive problems, calm the mind, improve sleep, nourish yin, and clear heat. |
| Huochuan |  | On the line joining Yangchi and the tip of elbow, 3 cun above the dorsocarpal transverse crease, between the ulna and radius. | It can relieve upper - limb pain, dredge the channels and collaterals, and treat disorders related to the heart and chest. |
| Wei (erzhen) | C04 | At the area where the crus of the helix disappears, specifically in the area 4 of the concha. | It can regulate stomach function, relieve gastric pain, control nausea and vomiting. |
| Pi(erzhen) | CO13 | In the upper - posterior part of the cavum conchae, at the mid - point of the line connecting the end of the crus of helix and the notch of the helix - tragus, in the area 13 of the concha. | It can regulate spleen function, enhance energy, alleviate fatigue, and regulat blood. |
| Yi(erzhen) | CO11 | At the edge of the cymba conchae, in the area between the auricular point of liver and the auricular point of duodenum. | It can regulate pancreatic function, aid in digestion, relieve pain associated with pancreatic disorders. |
| Neifenmi(erzhen) | CO18 | Within the intertragic notch, at the bottom of the cavum conchae. | It can regulate endocrine function, alleviate skin problems, and manage stress - related disorders. |
| Ji(erzhen) | HX6a､HX6b | At the mid - point of the lateral surface of the tragus. | It can regulate eating habits, suppress appetite, and manage  weight. |
| Ke(erzhen) |  | The "Thirst Point" is located on the outer side of the tragus. Specifically, it is at the midpoint of the line connecting the upper tubercle of the tragus and the root of the tragus. It is at the (slightly upper) midpoint of the line connecting the external nose and the tip of the tragus, and in the middle of the line connecting the external ear point and the hypertension point. | The "Thirst Point" is mainly used to treat symptoms such as neurogenic polydipsia, wasting - thirst, excessive thirst, diabetes, diabetes insipidus, dry mouth, and thirst. It has the function of promoting the production of body fluid and relieving thirst, can control the amount of water intake, and clear away the excessive pathogenic fire in the upper - jiao. It has a certain curative effect on wasting - thirst and excessive thirst caused by various reasons. In addition, the "Thirst Point" is also a reference point for diagnosing diabetes and diabetes insipidus. |
| sanjiao(erzhen) | CO17 | The Sanjiao acupoint is located at the bottom of the cavum conchae, behind and below the external auditory meatus, between the lung area and the endocrine area, above the endocrine acupoint, and in the middle of the line connecting the heart acupoint and the Zhixue 4 acupoint, that is, in the area 17 of the concha. | It can promote the downward - flowing of qi and help digestion, induce diuresis and resolve turbidity,  loosen the bowels and relieve pain,  nourish blood, and unblock menstruation |
| dan(erzhen) | C012 | It is located on the edge of the cymba conchae, specifically between the arc - shaped connection line of the liver point and the kidney point on the left ear. | Soothing the liver and promoting bile: It is helpful to regulate the functions of the liver and gallbladder and promote the secretion and excretion of bile.  Regulating qi to relieve pain: It can relieve the pain caused by stagnation of liver - qi or obstruction of gallbladder - qi.  Treating biliary diseases: Such as cholecystitis, gallstones, etc.  Improving ear symptoms: Such as tinnitus, deafness, etc.  Relieving migraine and neck rigidity: These symptoms are improved by regulating the qi and blood circulation of the gallbladder meridian. |
| sanjiao(erzhen) | CO17 | The auricular point "Sanjiao" (Triple Energizer) is located above the Endocrine point at the bottom of the concha cavity. | Regulating the endocrine: It can have an impact on the body's endocrine system and is helpful for maintaining the balance of the endocrine. For example, it has a certain regulatory effect on some symptoms caused by endocrine disorders.  Promoting water passage: It is helpful for the body's water - liquid metabolism and may play a certain role in improving conditions related to abnormal water - liquid metabolism such as edema and dysuria.  Regulating the zang - fu organs: From the overall concept of traditional Chinese medicine, Sanjiao is one of the six fu - organs and is closely related to other zang - fu organs. Stimulating this auricular point can play a role in regulating the functions of the zang - fu organs and has a certain auxiliary adjustment effect on some cases of zang - fu organ function disorders. |
| shenmen(erxue) | TF4 | Shenmen acupoint is located in the upper part of the posterior one - third of the triangular fossa, that is, area 4 of the triangular fossa. | 1.Sedation and Tranquilization It can be used to treat sleep - disorder - related diseases such as insomnia and dreaminess. By stimulating Shenmen acupoint, the function of the nervous system can be regulated, helping to relieve mental stress and making the body in a relaxed state, thus improving the quality of sleep.  2.Analgesia It has a certain relieving effect on various pains, such as headache and hypochondriac pain. It can regulate the qi and blood circulation in the body. When the qi and blood are unobstructed, the pain can often be alleviated.  3.Regulation of Endocrine It may have an auxiliary regulatory effect on some symptoms caused by endocrine disorders, such as irritability, anxiety and other emotional problems. Auricular Shenmen can affect the endocrine system through the nerve - endocrine - immune network, making the internal environment of the body tend to be stable. |
| bladder 38(fuyang) | BL59 | On the posterior side of the lower leg, posterior to the lateral malleolus, and 3 cun directly above Kunlun. It's approximately between the Achilles tendon and the fibula. | It can relieve pain and numbness in the lower limbs, treat lumbosacral pain, strengthen the lower extremities. |
| kidney 24(lingxu) | KI24 | In the chest region, in the third intercostal space, 2 cun lateral to the anterior | It can improve respiratory function,  relieve chest pain, and regulate emotion. |
