# Supplementary material for: The effect of acupuncture on blood glucose control in patients with type 2 diabetes: a systematic review and meta-analysis of randomized controlled trials
Source: Front Endocrinol (Lausanne). 2025 Jun 11;16:1596062. doi: 10.3389/fendo.2025.1596062 (PMC12187737; doi:10.3389/fendo.2025.1596062)
Supplement: Supplementary Table 3 — Distribution and Frequency of Acupuncture Points in Meridians Ranking Table [file Table3.docx]

**Table S3** Distribution and Frequency of Acupuncture Points in Meridians Ranking Table

| Ranking | Names of meridians | Acupoints ( frequency) | Number of acupoints | Total frequency of acupoints | Proportion of acupoints |
| --- | --- | --- | --- | --- | --- |
| 1 | Stomach Meridian of Foot Yangming | Neiting ( 5 ), Chongyang ( 2 ),  Zusanli ( 20 ), Shuidao ( 1 ),  Tianshu ( 5 ), Fenglong (7),  Jiexi (1) | 7 | 41 | 21.93% |
| 2 | Bladder Meridian of Foot Taiyang | Weiwanxiashu ( 2 ) , Ganshu ( 4 ) , Pishu ( 9 ) , Weishu ( 5 ) , Feishu ( 6 ) , Shenshu ( 8 ), Fuyang ( 1 ), Chengshan ( 1 ), Geshu ( 1 ) | 9 | 37 | 19.79% |
| 3 | Spleen Meridian of Foot Taiyin | Sanyinjiao ( 11 ), Yinlingquan ( 7), Daheng ( 2 ), Xuehai ( 3 ), Taibai (1) | 5 | 24 | 12.83% |
| 4 | Large Intestine Meridian of Hand Yangming | Quchi ( 9 ), Hegu ( 10 ), Jianyu ( 1 ) | 3 | 20 | 10.70% |
| 5 | Ren Meridian | Zhongwan (6) , Qihai (5) , Guanyuan (4), Chengjiang (1), Shuifen (1) | 5 | 17 | 9.10% |
| 6 | Auricular points | Sanjiao (1) , Hunger point (2) , Stomach (2) , Shenmen (1) , Endocrine (3) , Spleen (3), Thirst point (1), Jiaogan (1), Pancreas (2), Gallbladder (1) | 10 | 17 | 9.10% |
| 7 | Kidney Meridian of Foot Shaoyin | Taixi (5) , Rangu (1) , Zhaohai (3) | 3 | 9 | 4.81% |
| 8 | Liver Meridian of Foot Jueyin | Taichong (5), Xingjian (1), Ququan (2) | 3 | 8 | 4.28% |
| 9 | Sanjiao Meridian of Hand Shaoyang | Waiguan (3) , Jianliao (1), Zhigou (2), Yangchi (1) | 4 | 7 | 3.74% |
| 10 | Gallbladder Meridian of Foot Shaoyang | Yanglingquan (3) , Huantiao (1), Xiaxi (2) | 3 | 6 | 3.21% |
| 11 | Non-meridian extra acupoints | Bafeng (1) , Xiyan (1), Jinjin (1), Yuye (1) | 4 | 4 | 2.14% |
| 12 | Governor Vessel | Dazhui (2) | 1 | 2 | 1.07% |
| 13 | Lung Meridian of Hand Taiyin | Shaoshang (1) | 1 | 1 | 0.53% |
| 14 | Pericardium Meridian of Hand Jueyin | Neiguan (1) | 1 | 1 | 0.53% |
