# Supplementary material for: The effect of acupuncture on blood glucose control in patients with type 2 diabetes: a systematic review and meta-analysis of randomized controlled trials
Source: Front Endocrinol (Lausanne). 2025 Jun 11;16:1596062. doi: 10.3389/fendo.2025.1596062 (PMC12187737; doi:10.3389/fendo.2025.1596062)
Supplement: Supplementary Table 4 — Subgroup Analysis Results of the Impact of Acupuncture [file Table4.docx]

**Table S4** Subgroup Analysis Results of the Impact of Acupuncture

| Subgroup | Change in FBG | | | | Change in TG | | | | Change in HDL | | | | Change in 2h PG | | | |
| --- | --- | --- | --- | --- | --- | --- | --- | --- | --- | --- | --- | --- | --- | --- | --- | --- |
|  | Study | SMD [95%CI] | *P* value | *I*^2^ | Study | SMD [95%CI] | *P* value | *I*^2^ | Study | SMD [95%CI] | *P* value | *I*^2^ | Study | SMD [95%CI] | *P* value | *I*^2^ |
| **Total** | 22 | -0.35 [-0.50,-0.19] | ＜0.0001 | 63% | 11 | -0.16 [-0.32, -0.01] | 0.04 | 26% | 11 | 0.68 [0.29, 1.07] | 0.0007 | 91% | 11 | -0.31 [-0.49, -0.13] | 0.0008 | 55% |
| **Population** |  |  |  |  |  |  |  |  |  |  |  |  |  |  |  |  |
| Type 2 diabetes | 8 | -0.28 [-0.50, -0.05] | 0.02 | 39% | 1 | 0.02 [-0.48, 0.52] | 0.94 |  | 1 | -0.13 [-0.63, 0.37] | 0.62 |  | 1 | -0.05 [-0.57, 0.47] | 0.84 |  |
| Complications of type 2 diabetes | 9 | -0.38 [-0.62, -0.15] | 0.002 | 55% | 6 | -0.21 [-0.40, -0.02] | 0.03 | 7% | 6 | 1.09 [0.22, 1.95] | 0.01 | 95% | 6 | -0.20 [-0.39, -0.01] | 0.04 | 0% |
| Type 2 diabetes mellitus complicated with obesity | 1 | 0.30 [-0.34, 0.94] | 0.36 |  | 1 | 0.16 [-0.48, 0.80] | 0.63 |  | 1 | 0.87 [0.20, 1.53] | 0.01 |  | 0 |  |  |  |
| Prediabetes | 2 | -1.13 [-2.35, 0.09] | 0.07 | 85% | 2 | -0.54 [-1.12, 0.03] | 0.06 | 44% | 1 | 0.21 [-0.32, 0.74] | 0.43 |  | 1 | -0.98 [-1.67, -0.30] | 0.005 |  |
| Carbohydrate intolerance | 2 | -0.22 [-0.64, 0.20] | 0.31 | 88% | 1 | 0.00 [-0.23, 0.23] | 1.00 |  | 2 | 0.30 [0.11, 0.50] | 0.002 | 44% | 2 | -0.48 [-0.99, 0.03] | 0.07 | 92% |
| **Treatment duration** |  |  |  |  |  |  |  |  |  |  |  |  |  |  |  |  |
| ≥3 months | 15 | -0.35 [-0.54, -0.16] | 0.0002 | 71% | 8 | -0.21 [-0.41,-0.01] | 0.04 | 44% | 8 | 0.24 [0.12, 0.37] | 0.0002 | 12% | 10 | -0.32 [-0.51, -0.14] | 0.0005 | 57% |
| ＜3 months | 7 | -0.34 [-0.63, -0.05] | 0.02 | 37% | 3 | -0.05 [-0.36, 0.26] | 0.76 | 0% | 3 | 2.26 [0.81, 3.70] | 0.002 | 92% | 1 | 0.16 [-0.74, 1.06] | 0.73 |  |
| **Region** |  |  |  |  |  |  |  |  |  |  |  |  |  |  |  |  |
| China | 17 | -0.35 [-0.52, -0.17] | ＜0.0001 | 66% | 10 | -0.18 [-0.35,-0.02] | 0.03 | 28% | 10 | 0.66 [0.25, 1.08] | 0.002 | 91% | 11 | -0.31 [-0.49, -0.13] | 0.0008 | 55% |
| Non-China | 5 | -0.33 [-0.72, 0.05] | 0.09 | 58% | 1 | 0.16 [-0.48, 0.80] | 0.63 |  | 1 | 0.87 [0.20, 1.53] | 0.01 |  | 0 |  |  |  |
| **Mean/median age** |  |  |  |  |  |  |  |  |  |  |  |  |  |  |  |  |
| ≥50y | 14 | -0.35 [-0.53, -0.16] | 0.0002 | 56% | 4 | -0.29 [-0.61, 0.02] | 0.06 | 23% | 3 | 2.03 [0.06, 4.00] | 0.04 | 96% | 5 | -0.29 [-0.60, 0.02] | 0.06 | 31% |
| ＜50y | 8 | -0.34 [-0.64, -0.04] | 0.03 | 74% | 7 | -0.12 [-0.30, 0.06] | 0.20 | 27% | 8 | 0.26 [0.10, 0.42] | 0.001 | 37% | 6 | -0.31 [-0.55, -0.08] | 0.01 | 69% |
| **Acupuncture Methods** |  |  |  |  |  |  |  |  |  |  |  |  |  |  |  |  |
| Traditional acupuncture | 6 | -0.21 [-0.40,-0.02] | 0.03 | 0% | 1 | -0.29 [-0.82,0.24] | 0.29 |  | 1 | 0.21 [-0.32, 0.74] | 0.43 |  | 1 | -0.34 [-0.84,0.15] | 0.17 |  |
| Electroacupuncture | 4 | -0.42 [-0.82,-0.02] | 0.04 | 41% | 1 | 0.02 [-0.48,0.52] | 0.94 |  | 1 | -0.13 [-0.63,0.37] | 0.62 |  | 2 | -0.00 [-0.45,0.45] | 1.00 | 0% |
| Acupoint embedding thread | 2 | -0.85 [-2.60,0.89] | 0.34 | 95% | 2 | -0.38 [-1.23,0.47] | 0.38 | 83% | 1 | 0.41 [0.18,0.64] | 0.0004 |  | 2 | -0.77 [-0.99,-0.55] | ＜0.00001 | 0% |
| Special acupuncture therapy (balance needle, laser needle) | 2 | -0.13 [-0.83,0.57] | 0.03 | 78% | 1 | 0.16 [-0.48,0.80] | 0.63 |  | 2 | 0.46 [-0.16,1.08] | 0.15 | 71% | 1 | -0.22 [-0.40,-0.04] | 0.02 |  |
| Acupuncture plus drugs | 1 | -0.39 [-0.83,0.05] | 0.08 |  | 0 |  |  |  | 0 |  |  |  | 1 | -0.22 [-0.66,0.22] | 0.33 |  |
| Acupuncture at other parts， such as wrist and ankle acupuncture, body acupuncture, and auricular acupuncture | 6 | -0.49 [-0.79,-0.18] | 0.002 | 62% | 6 | -0.21 [-0.40,-0.02] | 0.03 | 7% | 6 | 1.09 [0.22, 1.95] | 0.01 | 95% | 4 | -0.19 [-0.40,0.02] | 0.08 | 0% |
