# Supplementary figures and images for: The effect of acupuncture on blood glucose control in patients with type 2 diabetes: a systematic review and meta-analysis of randomized controlled trials
Source: Front Endocrinol (Lausanne). 2025 Jun 11;16:1596062. doi: 10.3389/fendo.2025.1596062 (PMC12187737; doi:10.3389/fendo.2025.1596062)

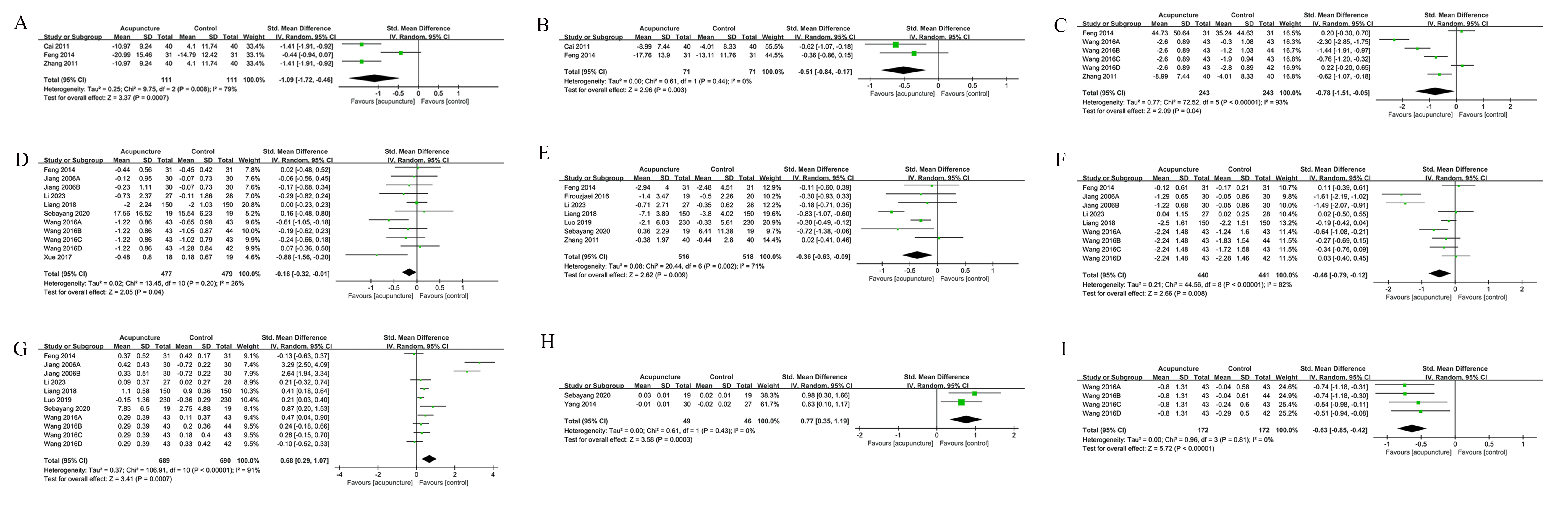

Supplement: Supplementary Figure 1 — (A) Forest plot FINS; (B) Forest plot Homa-IR; (C) Forest plot Homa-B; (D) Forest plot TG; (E) Forest plot BMI; (F) Forest plot LDL; (G) Forest plot HDL; (H) Forest plot WHR; (I) Forest plot Plasma Viscosity. [file Image1.tif]

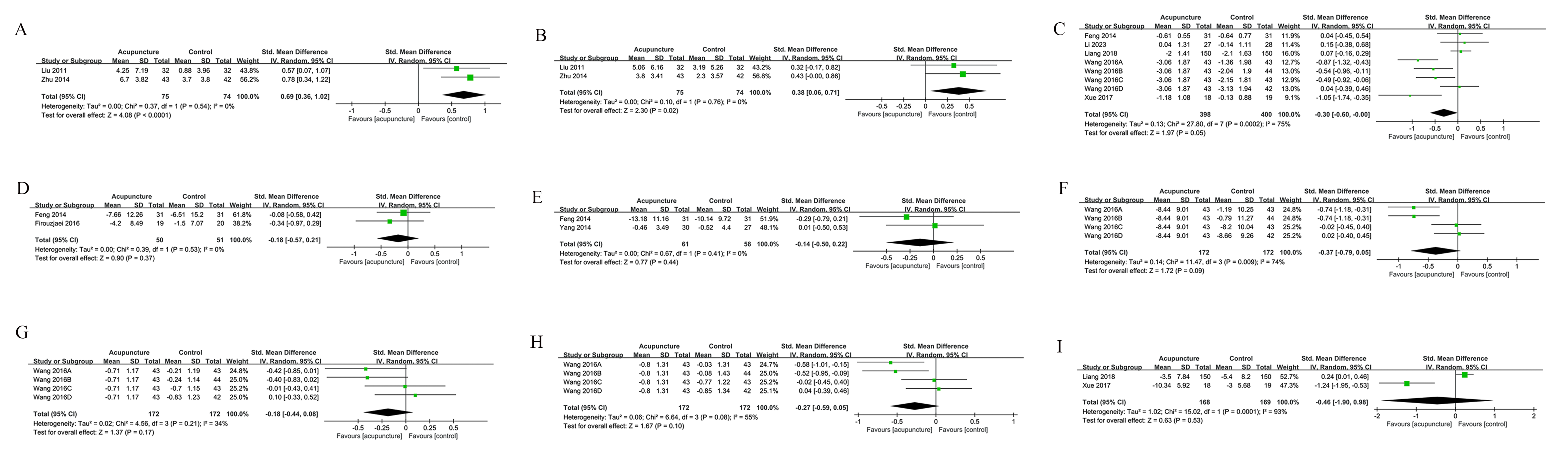

Supplement: Supplementary Figure 2 — (A) Forest plot Bilateral median nerve motor conduction velocity; (B) Forest plot Bilateral common peroneal nerve motor conduction velocity; (C) Forest plot TC; (D) Forest plot body weight; (E) Forest plot body fat percentage; (F) Forest plot PCV; (G) Forest plot whole blood viscosity; (H) Forest plot FIB; (I) Forest plot Scr. [file Image2.tif]

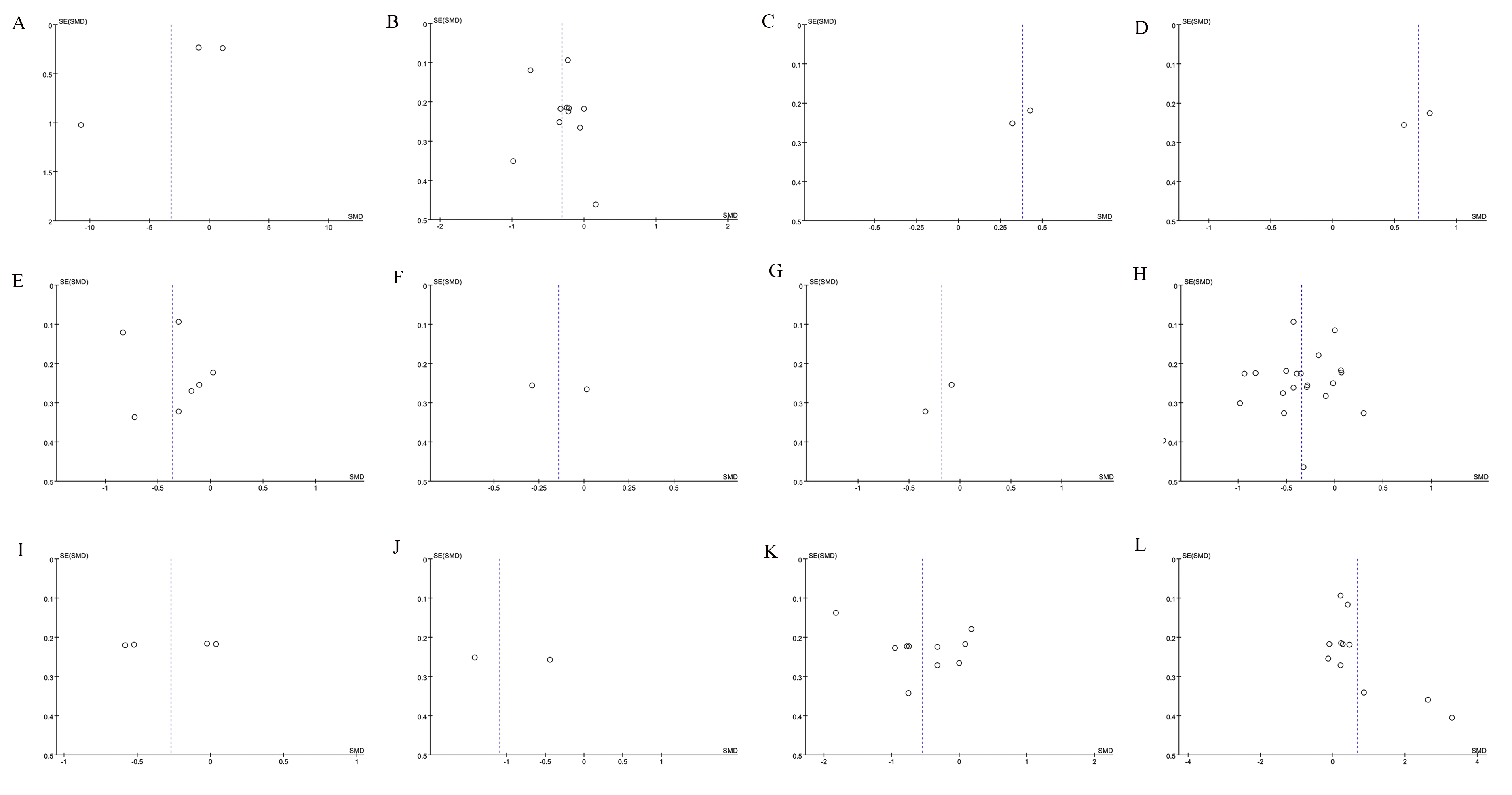

Supplement: Supplementary Figure 3 — (A) Funnel plot ISI; (B) Funnel plot 2h PG; (C) Funnel plot Bilateral common peroneal nerve motor conduction velocity; (D) Funnel plot Bilateral median nerve motor conduction velocity; (E) Funnel plot BMI; (F) Funnel plot body fat percentage; (G) Funnel plot body weight; (H) Funnel plot FBG; (I) Funnel plot FIB; (J) Funnel plot FINS; (K) Funnel plot HbA1c; (L) Funnel plot HDL. [file Image3.tif]

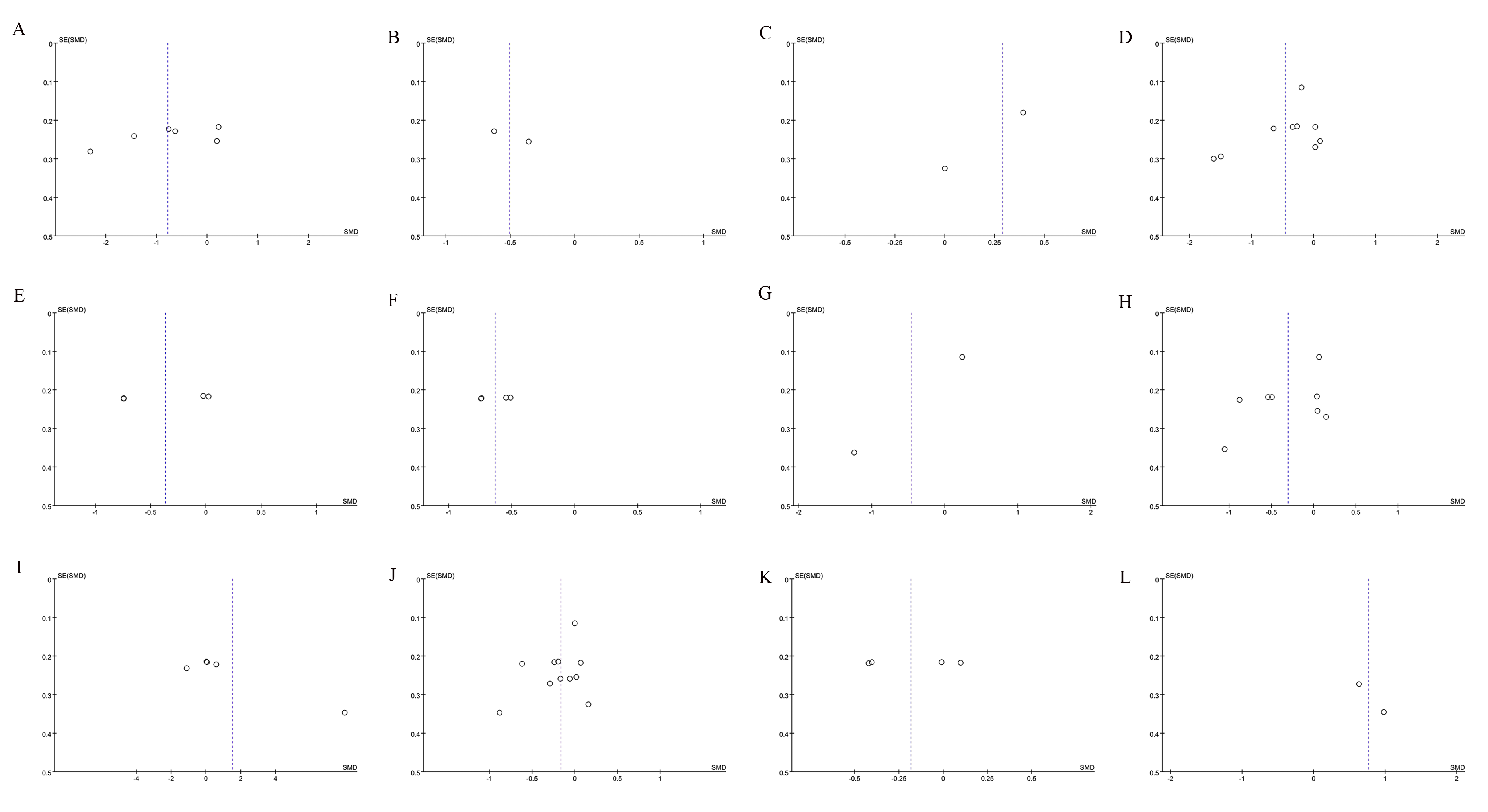

Supplement: Supplementary Figure 4 — (A) Funnel plot Homa-B; (B) Funnel plot Homa-IR; (C) Funnel plot Insulin levels; (D) Funnel plot LDL; (E) Funnel plot PCV; (F) Funnel plot Plasma Viscosity; (G) Funnel plot Scr; (H) Funnel plot TC; (I) Funnel plot TCMSS; (J) Funnel plot TG; (K) Funnel plot whole blood viscosity; (L) Funnel plot WHR. [file Image4.tif]

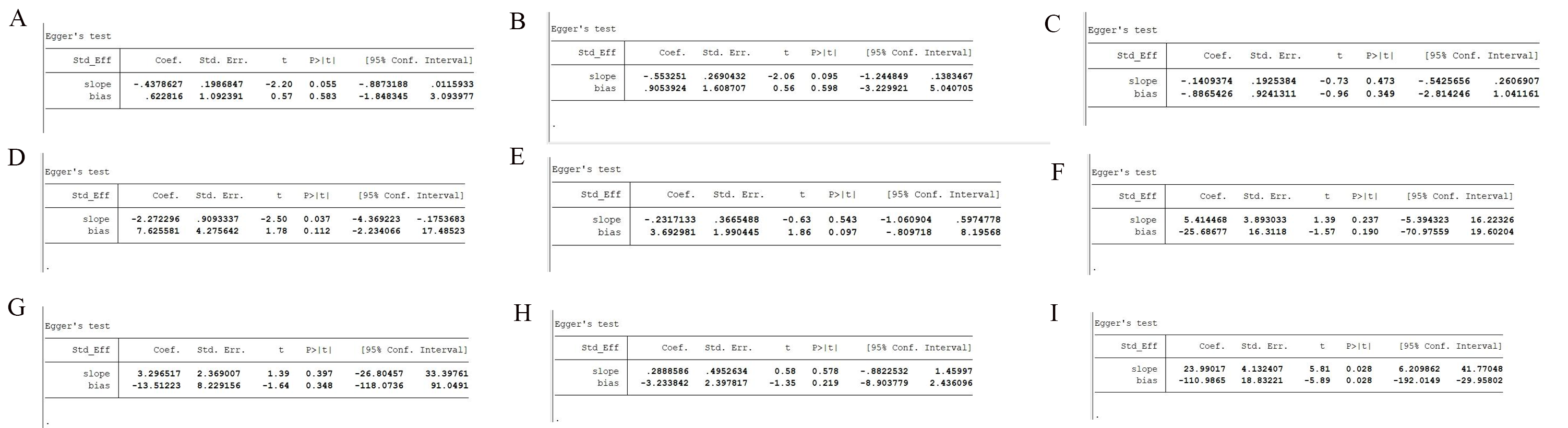

Supplement: Supplementary Figure 5 — (A) 2h PG; (B) BMI; (C) FBG; (D) glycated HbA1c; (E) HDL; (F) Homa-B; (G) ISI; (H) LDL; (I) PCV; [file Image5.tif]

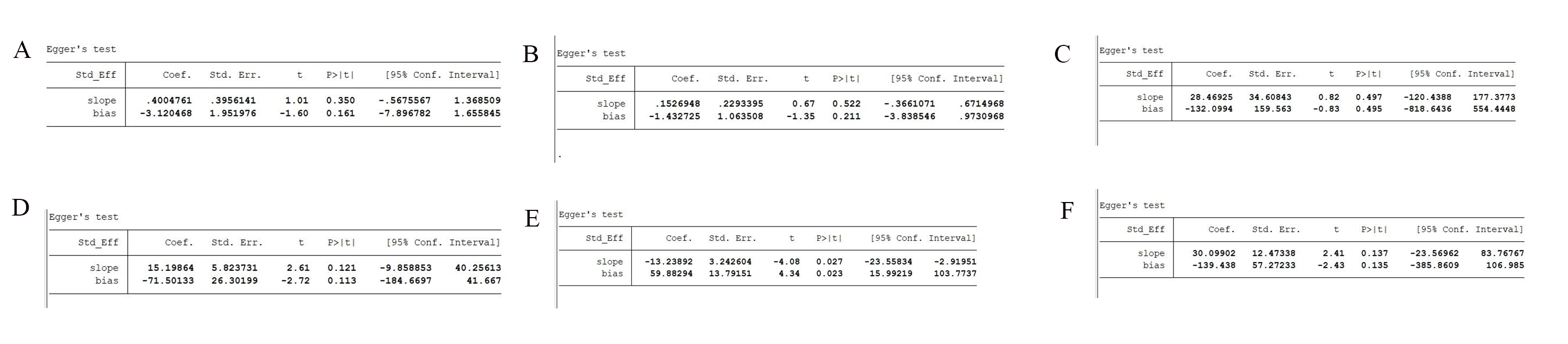

Supplement: Supplementary Figure 6 — (A) TC; (B) TG; (C) Whole Blood Viscosity; (D) Plasma Viscosity; (E) TCMSS; (F) FIB [file Image6.tif]

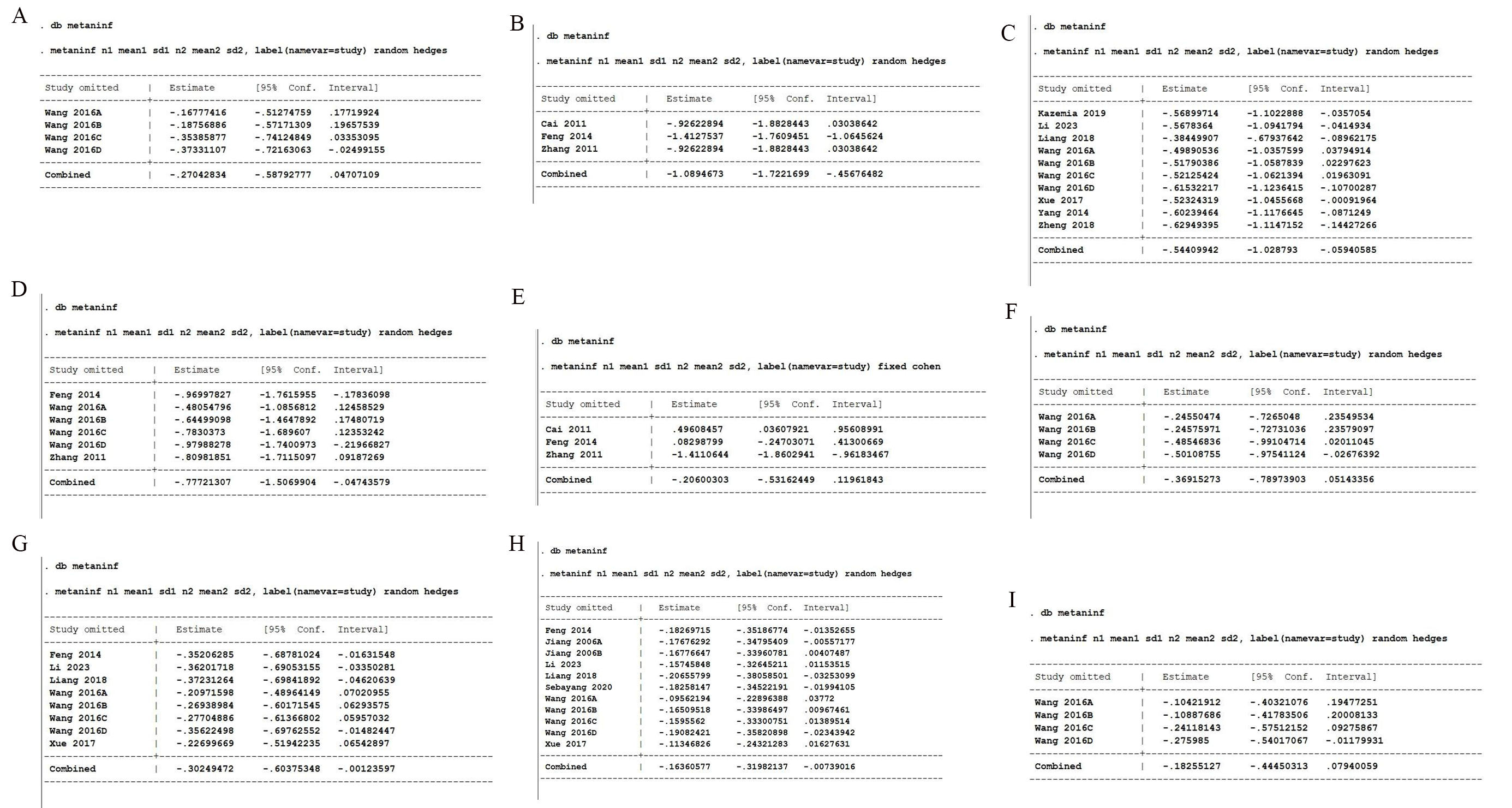

Supplement: Supplementary Figure 7 — (A) FIB; (B) FINS; (C) HbA1c; (D) Homa-B; (E) ISI; (F) PCV; (G) TC; (H) TG; (I) Whole Blood Viscosity [file Image7.tif]

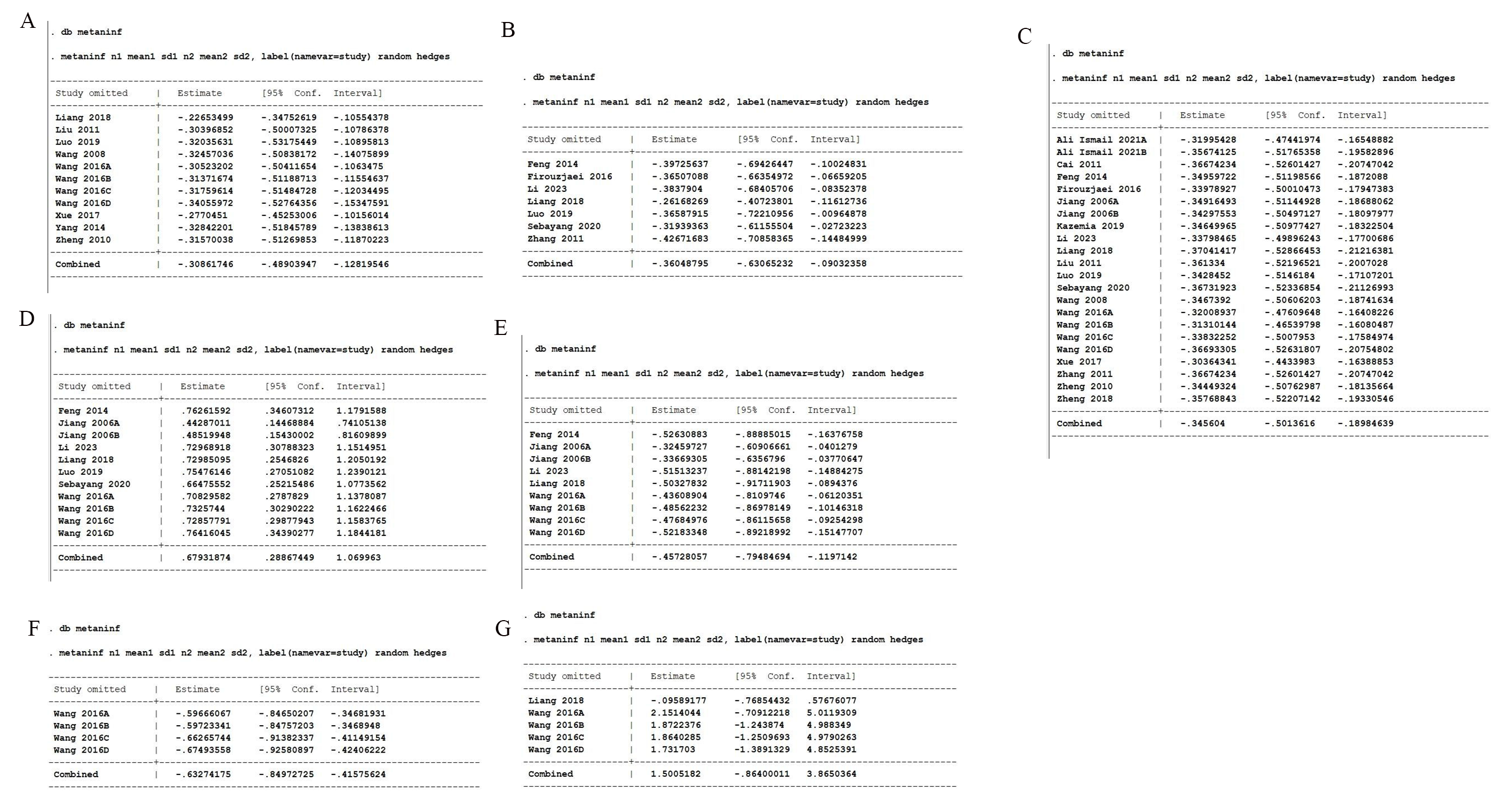

Supplement: Supplementary Figure 8 — (A) 2h PG; (B) BMI; (C) FBG; (D) HDL; (E) LDL; (F) Plasma Viscosity; (G) TCMSS [file Image8.tif]
